# Pharmacological and Non-Pharmacological Interventions to Improve Sleep in People with Cognitive Impairment: A Systematic Review and Meta-Analysis

**DOI:** 10.3390/ijerph22060956

**Published:** 2025-06-18

**Authors:** Patrick Crowley, Mark R. O’Donovan, Peter Leahy, Evelyn Flanagan, Rónán O’Caoimh

**Affiliations:** 1 Health Research Board Clinical Research Facility, University College Cork, Mercy University Hospital, T12WE28 Cork City, Ireland; pc420777@muh.ie (P.C.); markodonovan@ucc.ie (M.R.O.); e.flanagan@ucc.ie (E.F.); 2 Department of Respiratory and Sleep Medicine, Mercy University Hospital, T12WE28 Cork City, Ireland; pl417796@muh.ie

**Keywords:** sleep, mild cognitive impairment, dementia, systematic review, meta-analysis, treatment, pharmacological, non-pharmacological

## Abstract

Sleep disturbance is common among people with cognitive impairment and, when present, is an important target for intervention because it potentially leads to negative outcomes and cognitive decline. Given this association, sleep represents a potential public health target, although evidence for efficacy is lacking. For this study, a systematic review and meta-analysis was undertaken of controlled clinical trials of pharmacological and non-pharmacological interventions to improve sleep in mild cognitive impairment and dementia. A total of 144 trials involving 13,471 participants (median 50 per trial) were included. To measure sleep, 68 trials used subjective measures exclusively, and 41 used only objective measures, while 35 used a combination. In all, 287 discrete sleep outcome measures were reported. Bright light therapy was the most frequently examined non-pharmacological intervention, but results were equivocal. Other non-pharmacological interventions (such as physical activity, cognitive behavioural therapy for insomnia, music, and continuous positive airway pressure) showed promise but require further evidence. Results for melatonin, the most frequently examined pharmacological intervention, were inconclusive, but lower doses may be more effective. Other pharmacological interventions (such as trazadone and orexin-receptor antagonists) demonstrated effectiveness in a small number of trials but require further evidence. Overall, there is insufficient evidence upon which to base clinical decisions regarding the treatment of sleep disturbance in this population. Existing research is marked by wide heterogeneity, which limits opportunities for data synthesis. A core outcome set is urgently required to ensure that future research provides more coherent and reliable evidence to improve outcomes for people with cognitive impairment and sleep disturbance.

## 1. Introduction

There is a well-established association between sleep disturbance and cognitive impairment [1,2]. Studies have shown that, compared to healthy age-matched controls, people with mild cognitive impairment (MCI) experience reduced total sleep time, sleep efficiency, and duration of rapid eye movement (REM) sleep, whilst sleep onset latency, wakefulness after sleep onset, and duration of stage one sleep are increased [3,4]. Evidence that sleep becomes altered prior to the development of cognitive symptoms has raised the prospect that sleep disturbance could serve as a biomarker for the development of cognitive impairment [5,6], with emerging evidence that the relationship between sleep disturbance and cognitive impairment may be causal and bi-directional [6,7,8]. There is, for example, plausible evidence that sleep disturbance plays a role in the accumulation of beta-amyloid and tau, which are implicated in the pathophysiology of Alzheimer’s Disease (AD) [9]. Cerebro-spinal fluid (CSF) levels of beta-amyloid fluctuate diurnally, rising during wakefulness before declining during sleep [10]. The glymphatic system that clears such toxins from the brain is more efficient during sleep [11]. Sleep deprivation has been shown to increase the accumulation and cortical deposition of both beta-amyloid [12] and tau [13]. A recent meta-analysis of observational studies estimated that 15% of AD may be attributable to sleep disturbance [14]. Preventing or delaying dementia is a public health priority [15]. Given the relationship between sleep disturbance and cognitive decline, sleep would seem a reasonable target in this population, particularly as sleep interventions are often simple, relatively easy to implement, and widely practicable. Although the Dementia Prevention, Intervention and Care: 2024 report of the Lancet Commission has reaffirmed that further research is needed to clarify the effect of sleep disturbance on cognitive decline [15], interest is nevertheless growing in sleep disturbance as a modifiable risk factor for cognitive impairment [16].

Sleep disturbance tends to worsen as cognitive impairment advances [17,18], leading to deteriorating cognitive outcomes [19] and quality of life [20], as well as strained relationships with caregivers who experience considerable physical and emotional distress and burnout as a consequence [21,22]. The increased caregiver burden caused by sleep disturbance in people with dementia has been shown to lead to sub-optimal care [23] and early institutionalisation [24,25]. Sleep is therefore an important treatment target throughout the natural history of dementia, to potentially slow the progression of early-stage disease and to reduce the burden of symptoms in more advanced cases. Furthermore, sleep disturbance is common among this population, with one review estimating that up to 70% of people with cognitive impairment are affected [19]. There are approximately 55 million people living with dementia worldwide, and, with a rapidly growing and ageing population, its global prevalence is expected to increase to 139 million by 2050 [26]. Unfortunately, despite the recent emergence of disease-modifying therapies [27,28], there remains no curative treatment for dementia. Clinical management, therefore, remains largely focussed on addressing modifiable risk factors and reducing the burden of symptoms. In this context, there is a growing imperative to define the existing evidence base for interventions to improve sleep in people with cognitive impairment and to identify gaps in the literature where further research is required.

It is recognised, however, that ‘good sleep’ is a challenging concept to define and measure [29,30], particularly among people with cognitive impairment [31]. Furthermore, in addition to measuring sleep per se, it is important to consider the many ways in which sleep affects other aspects of health and well-being [32,33]. A previous scoping review of the academic literature involving the measurement of sleep among people with MCI and mild dementia demonstrated wide heterogeneity both in the manner in which sleep was measured and in the outcome measures reported [29]. In addition to raising the likelihood of reporting bias, this heterogeneity in reported outcome measures limits opportunities for data synthesis, which is particularly problematic when most of the clinical trials in this field involve small sample sizes [34]. The aforementioned scoping review did not include research involving participants with moderate-to-severe dementia and included only measures of sleep. It did not include outcome measures involving the effects of sleep on other aspects of health and well-being. Other literature reviews have been similarly limited in scope, either being restricted to pharmacological [35,36] or non-pharmacological [34,37,38] interventions or being restricted to particular stages [39] or aetiologies [35] of cognitive impairment.

This systematic review and meta-analysis, therefore, aims to (a) interrogate the evidence base from clinical trials of both pharmacological and non-pharmacological interventions to improve sleep in people with MCI and dementia and (b) delineate the outcome measures used in those trials in order to inform the development of a core outcome set [40] that will help ensure that future clinical trials are not beset by the heterogeneity that has been characteristic of the research undertaken heretofore.

## 2. Materials and Methods

The protocol for this systematic review and meta-analysis has been published elsewhere [41] and was prospectively registered on the International Prospective Register of Systematic Reviews (PROSPERO), registration number: CRD42024556750. A brief summary of the eligibility criteria, search strategy, and study selection process is outlined in the following paragraphs.

### 2.1. Eligibility Criteria

This systematic review and meta-analysis included controlled clinical trials that assessed interventions to improve sleep in people with MCI or dementia. Studies were included if they were original peer reviewed articles, written in the English language, reporting on clinical trials of interventions to improve sleep in participants with MCI or dementia. Studies were included if at least 80% of participants had a stated diagnosis of MCI or dementia, or, if no such diagnosis was stated, at least 80% of participants had to score within the generally accepted threshold for cognitive impairment on standard neurocognitive testing or, if said percentage was impossible to calculate based on the information available, the standard deviation/interquartile range of scores on standard neurocognitive testing had to be within the generally accepted threshold for cognitive impairment. Studies were also included if a distinct sub-group of participants, for whom results were separately reported, met this inclusion criterion. Included studies had to provide a comparison of the studied intervention with at least one other intervention/non-intervention/placebo control group. While cross-over trials were included, within-subjects trials that consecutively compared the studied intervention with another intervention or control within the same cohort of participants were not included. Finally, at least one primary or secondary outcome relating to sleep had to be reported. Studies that used composite assessments involving sleep as outcome measures were included only if the sleep component of the assessment was separately reported. Studies primarily involving participants who were post-stroke or post-traumatic brain injury, in-patients in intensive care units, or oncology patients were excluded. Grey literature, including abstracts/conference proceedings and book chapters, was not included. A summary of the eligibility criteria is provided in Table 1.

### 2.2. Search Strategy

The following databases were searched from inception to 3 October 2023: Medline (Ovid), CINAHL, PsycINFO, and the Cochrane CENTRAL database. This was the date finalised in the published protocol [41]. The detailed search strategy for each database is reported in the protocol and summarised in Supplementary Materials (Table S1) [41]. Broadly, the search strategy involved the following Boolean expression: ‘sleep’ (plus synonymous terms) AND ‘[cognitive impairment (plus synonymous terms) OR nursing home resident (plus synonymous terms)]’ AND ‘clinical trial’. The interTASC Information Specialists Sub-Group (ISSG) Search Filter Resource was used to filter for clinical trials [42]. Given the high prevalence of cognitive impairment among nursing home residents [43,44], it was felt prudent to include ‘nursing home resident’ (and synonymous terms) with ‘cognitive impairment’ (and synonymous terms) in order to ensure the capture of trials conducted in nursing homes that did not explicitly reference participants’ cognitive diagnoses but may nevertheless meet our inclusion criteria by reporting cognitive assessment scores. To illustrate this point, in a 2018 systematic review of non-pharmacological interventions to improve night-time sleep among residents of long-term care settings [45], over half of included studies involved participants with dementia but this was not always explicitly stated in the studies themselves [46]. The search strategy was reviewed in accordance with the Peer Review of Electronic Search Strategies (PRESS) Guideline Statement [47].

### 2.3. Study Selection

**Figure 1:**
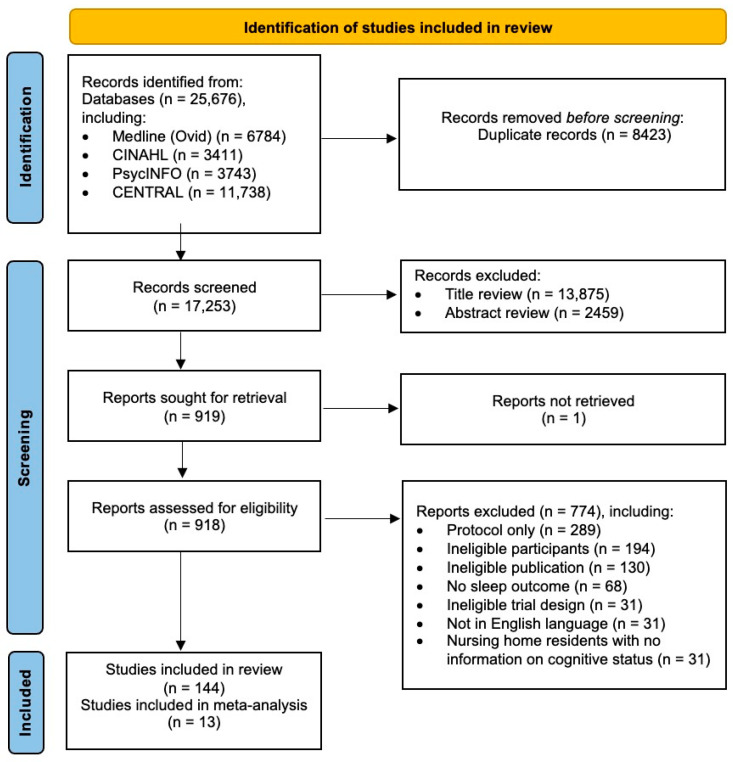
PRISMA flowchart showing studies included in the systematic review and meta-analysis.

The Preferred Reporting Items for Systematic Reviews and Meta-Analyses (PRISMA) guidelines were followed [48]. All identified citations were collated using reference management software (Endnote version 21), and duplicates were removed. Study titles were reviewed, and those that were clearly irrelevant were removed. Remaining abstracts were then reviewed and, again, only those that clearly did not meet the inclusion criteria were removed. The full texts of all remaining studies were then reviewed independently by two separate reviewers [P.C. and P.L.]. Discrepancies were resolved by consensus following discussion and, if required, adjudicated by the principal investigator [R.O.C.]. Reasons for the removal of studies at this stage were recorded. Where more than one paper described the same trial, the paper with the most complete data was included, such as the paper with the largest sample size or the longest period of follow-up, in that order of hierarchy. The study selection process is illustrated in the PRISMA flow diagram [49] (Figure 1).

### 2.4. Quality and Risk of Bias Assessment

The methodological quality of included trials was evaluated using a combination of the Joanna Briggs Institute Checklists for Quasi-experimental and for Randomised Controlled Trials [50]. Risk of bias was assessed using the Cochrane Risk of Bias 2 (RoB2) tool [51]. Both methodological quality and risk of bias were assessed independently by two reviewers [P.C. and P.L.], with any discrepancies resolved by a third reviewer (R.O.C).

### 2.5. Data Extraction

Data extraction was performed by two independent reviewers (P.C. and P.L.) using a pre-defined form (see protocol [41]).

### 2.6. Data Synthesis and Analysis

A narrative summary of the data from included trials is presented. The high risk of bias inherent in many of the included trials, together with the heterogeneity of reported outcome measures, precluded the statistical synthesis of data from most of the trials included in this review. After excluding trials that were adjudged to have a high risk of bias, random effects meta-analyses were performed when data were available from three or more trials involving a particular intervention modality or if data were available from the only two trials included in the review with respect to a particular intervention modality. Heterogeneity associated with clinical and/or methodological diversity was assessed using the chi-squared test and I^2^ statistic [52] (with values ≥ 70% indicating large heterogeneity). Funnel plots were used to assess publication bias.

## 3. Results

### 3.1. Study Selection

In total, 144 trials involving 13,471 participants were included. The median number of participants per trial was 50. In all, 79 of the included trials involved community-dwelling participants exclusively, while 45 involved only residents of nursing homes or assisted living facilities, and 14 involved hospital in-patients. Additionally, three trials involved both nursing home residents and community-dwellers, whilst two trials involved a combination of nursing home residents and hospital in-patients. One further trial involved a combination of community dwellers and hospital in-patients. The characteristics of the included trials are set out in the Supplementary Materials (Table S2). This provides a description of the experimental results, their interpretation, as well as the experimental conclusions that can be drawn. Please note, all of the trials included in Table S2 are referenced in chronological sequence as if Table S2 appeared at this point in the text.

### 3.2. Quality and Risk of Bias Assessment

The results of the assessments of methodological quality and risk of bias are set out in the Supplementary Materials (Table S3). Overall, just over half (80/144, 55.56%) of the included trials were adjudged to be at high risk of bias, primarily due to a loss of participants to follow-up without an adequate explanation of the reasons for same.

### 3.3. Sleep Measurement Tools

**Figure 2:**
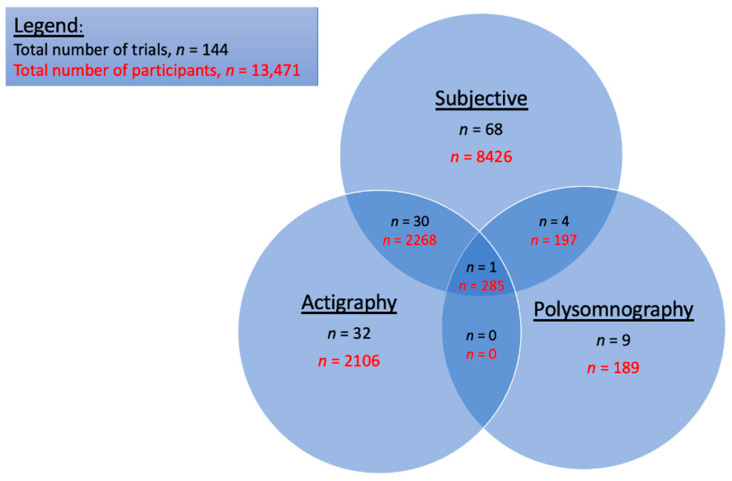
Number of trials and participants per method of sleep measurement.

Of the 144 included trials, 103 used subjective measurement tools to assess sleep outcome measures. Of these, 30 were in combination with actigraphy, four added polysomnography, and one combined both actigraphy and polysomnography. In total, actigraphy was used in 63 trials, whilst only 14 trials used polysomnography to measure sleep outcomes. Figure 2 provides a breakdown of the number of trials and the number of participants involved in those trials, with respect to each of these different methods of sleep measurement, namely actigraphy, polysomnography, and subjective measures.

### 3.4. Sleep Outcome Measures

**Figure 3:**
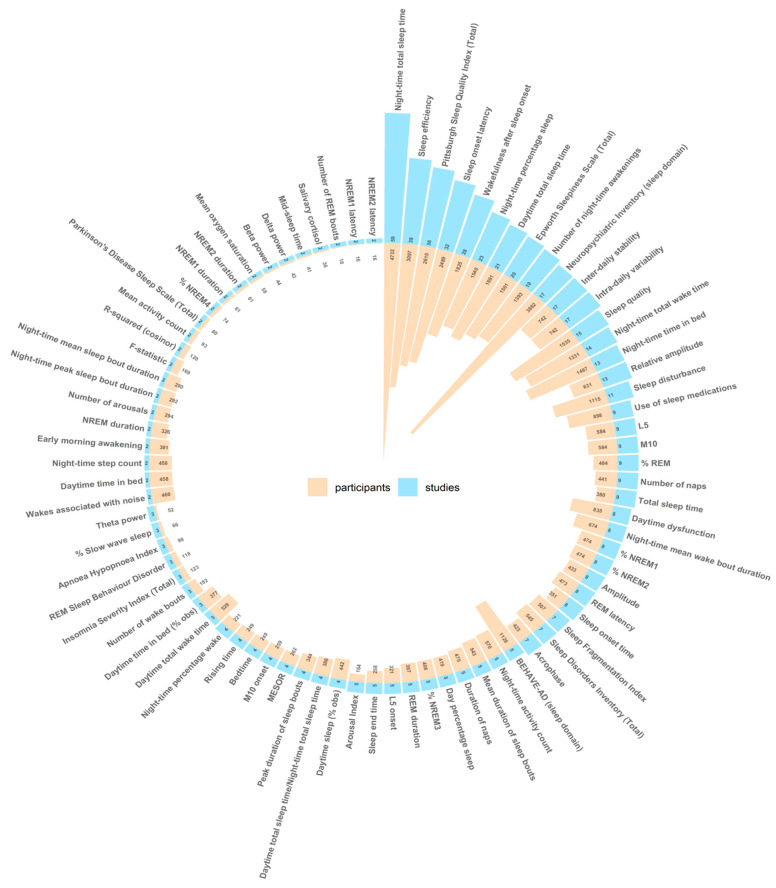
The sleep outcome measures reported in more than one trial (*n* = 82) including the total number of studies including each outcome and participants involved in those trials.

In total, across the 144 included trials, 287 discrete sleep outcome measures were reported. Of these outcomes, 205 appeared in only one trial, while a further 23 appeared in only two trials. Figure 3 presents the sleep outcome measures that were reported in more than one trial (*n* = 82) and illustrates the number of trials in which each sleep outcome measure was reported with the total number of participants involved in those trials. The complete data, including data pertaining to the 205 outcome measures that appeared in only one trial, are provided in the Supplementary Materials in Table S4 and graphically in Figure S2.

The most commonly used outcome measures were night-time total sleep time (59 trials involving 4732 participants) and sleep efficiency (39 trials involving 3097 participants). In all, 23 of the reported sleep outcome measures involved scores of recognised questionnaires. The Pittsburgh Sleep Quality Index (PSQI) [53] was the most commonly used questionnaire, being reported in 36 trials involving 2610 participants. The sleep and night-time behaviour disorders section of the Neuropsychiatric Inventory (NPI) [54], however, was the most commonly used by participant number, being used in 17 trials involving 3882 participants. Other commonly used questionnaires included the Epworth Sleepiness Scale (ESS) [55] (20 trials involving 1501 participants) and the Behavioural Pathology in Alzheimer’s Disease Rating Scale (BEHAVE-AD) [56] (five trials involving 1128 participants). In total, 16 of the 23 questionnaires were used in only one trial. Therefore, no single outcome measure was used in over half of the included trials.

### 3.5. Other Non-Sleep Outcome Measures

Just over half of all included trials (75/144) reported outcome measures relating to cognition. The Mini-Mental State Examination (MMSE) [57] was the most commonly used cognitive instrument (51 of the 75 trials). The Alzheimer’s Disease Assessment Scale—Cognitive Subscale (ADAS-Cog) [58] was used in 18 trials, while the Montreal Cognitive Assessment (MoCA) [59] was used in 14. In all, 55 trials reported outcome measures relating to behavioural disturbance. The most commonly used outcome measures in this regard were the NPI [54], which was used in 31 trials, followed by the Cohen-Mansfield Agitation Inventory [60], which was used in nine trials, and the BEHAVE-AD [56], which was used in six trials. We identified 48 trials reporting outcome measures relating to mood, the most common being the Cornell Scale for Depression in Dementia [61], used in 13 trials, and the Geriatric Depression Scale [62], used in 12 trials. Functional ability was reported as an outcome measure in 31 trials. The most commonly used instruments in this regard were the Alzheimer’s Disease Co-operative Study-Activities of Daily Living (ADCS-ADL) Scale [63], which was used in seven trials, and the Katz Index of Independence in Activities of Daily Living [64], which was used in six trials. Quality of life was reported as an outcome measure in 12 trials, with the most commonly employed measurement instruments being the EQ-5D-5L [65], used in three trials, and the Quality of Life in Alzheimer’s Disease (QoL-AD) [66], also used in three trials. In total, 10 trials reported outcome measures relating to physical activity. Two trials reported the effect of an intervention on neurodegenerative biomarkers, and one trial reported changes on brain imaging (MRI). We found that 19 trials reported pre- and post-intervention outcomes relating to the effect on carers, including three trials that reported solely on caregivers’ sleep, nine that reported on other aspects of caregivers’ well-being, and seven that reported both on caregivers’ sleep and other aspects of their well-being.

### 3.6. Interventions (Non-Pharmacological and Pharmacological)

The majority, 95 of the 144 (66%) trials, involved non-pharmacological interventions, while 46 of the 144 (32%) trials examined pharmacological interventions. Three additional trials examined pharmacological interventions as part of a multi-modal intervention that also incorporated non-pharmacological elements. A narrative summary of the results of the trials with respect to each intervention will be set out in the following paragraphs, with the results of meta-analyses described where these were undertaken. Forest plots illustrating the results of meta-analyses undertaken are provided in the Supplementary Materials (Figure S1a–i).

#### 3.6.1. Non-Pharmacological Interventions

##### Bright Light Therapy

Although bright light therapy (BLT) was a component of several multi-modal interventions, 23 trials that reported the effects of BLT alone were included. Of these, 18 involved hospital in-patients or residents in nursing homes or assisted living facilities. None of the five remaining trials that involved community-dwelling participants produced any significant change in objective measures of sleep, except for one trial that significantly reduced night-time total wake time [67]. Results overall were markedly heterogeneous. Nine trials did not produce any significant effects on sleep parameters [68,69,70,71,72,73,74,75,76], although three of these did demonstrate improvements in circadian rhythmicity [72,73,75]. Indeed, three trials demonstrated a significant deterioration in sleep parameters in the intervention groups [77,78,79], although, again, one of these produced an improvement in rhythmicity [78]. Of the 11 trials that demonstrated significant improvements in sleep, three used only subjective sleep measures [80,81,82], while another three trials produced significant improvements in subjective sleep quality but failed to show any significant improvement in objectively measured sleep parameters [83,84,85]. Overall, only four trials demonstrated significant improvement in sleep parameters using an objective measurement tool [67,86,87,88], but, while one of these showed a significant increase in the maximum sleep bout duration, there was no overall improvement in total sleep time [86]. Another trial examining BLT showed a significant reduction in night-time activity levels only in those with vascular dementia, not in those with Alzheimer’s Disease [89].

Meta-analysis did not show any significant effect of BLT on either night-time total sleep time (mean difference: 6.02; 95% CI: −27.97–40.02; I^2^ = 0%) or sleep efficiency (mean difference: 1.32; 95% CI: −9.60–12.25; I^2^ = 62.5%). These results accord with two earlier systematic reviews that concluded that there is insufficient evidence to support BLT as a treatment for sleep in people with cognitive impairment [90,91].

##### Multi-Modal Interventions

Eleven trials evaluated multi-modal interventions. Six of these involved nursing home residents, while five involved community-dwelling participants. The interventions were heterogeneous in composition but mainly involved BLT, physical exercise, bed restriction, medication, sleep hygiene education, nursing, and noise-related interventions. While five trials [67,88,92,93,94] achieved significant improvements in night-time sleep, one of these did not produce a significant improvement in objective parameters whilst improving subjective sleep quality [93]. Of the remaining six trials that did not achieve any significant improvement in night-time sleep [95,96,97,98,99,100], four did produce significant improvements in daytime sleep/sleepiness/time in bed [95,96,98,99]. Only three trials assessed the effect of the multi-dimensional intervention on cognition [88,93,94], with none finding any significant improvement. It was not possible to assess the effectiveness of multi-modal interventions using meta-analysis.

##### Sensory Stimulation

In total, twelve trials examined the effect of various forms of sensory stimulation on sleep in persons with cognitive impairment. Results from the three trials examining transcutaneous electrical stimulation were generally promising, with two showing improvement in inter-daily stability [101,102], whilst the other found improvement in subjective sleep quality and cognition [103].

Four trials examined transcranial electrical stimulation. One of these trials used polysomnography to analyse the effect of transcranial direct current stimulation applied during an afternoon nap [104]. It found a significant enhancement of frontal and centroparietal cortical slow oscillation power and fast spindle power, with a statistically significant enhancement of frontal slow spindle power. There was also improved slow oscillation and spindle synchronisation. While there was significant improvement in visual memory, there was no significant improvement in verbal memory, procedural memory, or location memory. Stimulation-induced improvements in visual memory were correlated with enhanced synchronisation between slow oscillations and fast spindle power, suggesting that such enhanced synchronisation may play a role in mediating the improvements in visual memory consolidation. The other three trials involving transcranial electrical stimulation, which used methods of sleep measurement other than polysomnography, did not find any significant improvements in sleep or rest–activity rhythms [105,106,107].

Transcranial magnetic stimulation achieved significant improvements in subjective sleep quality and cognition in one trial [108], while in another trial the combination of transcranial magnetic stimulation and transcranial electrical stimulation produced significant improvements in subjective sleep quality, cognition, and neuropsychiatric symptoms [109]. A transcutaneous infra-red light helmet device produced significant improvements in cognition [110]. Acoustic stimulation produced a significant enhancement in cortical slow oscillations and slow wave activity, but, while a trend towards improvement in cognition was found, it did not reach statistical significance [111]. A combination of visual and auditory stimulation produced a significant reduction in night-time active duration [112]. It was not possible to assess the effect of sensory stimulation interventions using meta-analysis.

##### Physical Activity

Eleven trials examining the effects of physical activity on sleep were included. These included different activities ranging from dynamic sitting [113] to stepping [114], sit-to-stand repetitions [46], structured limb exercise [115], walking [67,116] with the addition of rhythmic exercises [117], outdoor activities involving gardening [118], tailored activity programmes [119], and more extensive multi-modal exercise programmes involving aerobic, flexibility, resistance, and balance training [120,121]. Seven of these trials showed statistically significant improvements in sleep [67,113,114,115,117,118,120]. All five trials that assessed cognition pre- and post-intervention found significant improvements [113,114,115,116,117], including one trial involving walking programmes of varying intensity that did not find a concurrent improvement in sleep [116]. Sleep was not the primary outcome in any of the other four trials that found no significant improvement in sleep parameters [46,116,119,121]. Two of these were secondary analyses of trials designed to improve physical endurance/performance [46,121], while another assessed cognition as the primary outcome and only assessed sleep for its mediating effects [116]. In the remaining trial, only the sleep domain of the NPI was included as part of a wider assessment of neuropsychiatric disturbances [119]. Again, a meta-analysis was not possible.

##### Continuous Positive Airway Pressure (CPAP)

Seven trials examined the effects of CPAP. All involved community dwelling participants. Three trials that assessed night-time sleep parameters demonstrated significant improvements in sleep [122,123,124]. One of these trials used polysomnography [122], whilst the other two used the PSQI to measure subjective sleep quality [123,124]. While two trials showed improvements in daytime sleepiness with CPAP in persons with cognitive impairment [125,126], one did not [127]. All five trials that assessed the effect of CPAP on cognition found significant improvements [123,124,127,128,129]. Two of these trials also found significant improvements in mood [123,124], whilst one showed a significant improvement in blood-based biomarkers of neurodegeneration [124].

##### PARO Robotic Seal

PARO Robotic Seal is an advanced interactive therapeutic robot designed to stimulate people with dementia [130]. All four trials that examined the effect of PARO Robotic Seal took place in nursing homes. Only one trial found a significant improvement in overnight sleep parameters [131]. None of the other three trials achieved any significant improvement in night-time sleep parameters [132,133,134], despite one of these trials producing a reduction in daytime sleep and an increase in daytime total wake time [133].

##### Acupressure and Massage

Two trials assessed the effect of massage. One of these found no significant effect on sleep [135], while the other only found a statistically significant improvement in the sleep quality sub-domain of the PSQI without significant improvement in the overall score [136]. One trial independently examined the effect of both acupressure and massage and found both significantly improved sleep [137].

##### Tai Chi

Three trials examined the effect of Tai Chi. One of these trials involved 60-min-long group sessions of Tai Chi Qigong twice per week for two months [138]. The other two trials involved Yang style Tai Chi [139,140]. One of these latter trials incorporated three group sessions of Tai Chi and two group sessions of routine exercise per week for 12 weeks, with each session lasting 40–50 min [140]. The other trial involved 39 Tai Chi sessions of 40–45 min duration three times per week for 13 weeks [139]. All three trials took place in China and found significant improvements in subjective sleep quality. The two trials involving Yang style Tai Chi, both of which involved patients with co-morbid Parkinson’s Disease, also demonstrated significant improvements in cognition [139,140]. It is notable, however, that one of these trials compared group sessions of Tai Chi with individual sessions of Tai Chi and only the former demonstrated improvements in cognition, suggesting that, perhaps, the social aspect of the group sessions may have been a confounding factor in this regard [139]. The trial involving Tai Chi Qigong did not show any significant improvement in cognition [138]. There was no significant improvement in mood [139,140] or quality of life [138,140].

##### Cognitive Behavioural Therapy for Insomnia (CBT-I)

Two trials examined the effect of CBT-I, and both found significant improvements in sleep [141,142]. One trial involving 28 participants from independent living facilities achieved significant improvement in both subjective and objective measures of sleep using the Insomnia Severity Index and wrist actigraphy [141]. The other trial, involving 35 participants recruited from outpatient clinics, found no improvement in objective measures, with significant improvements only in subjective sleep quality [142]. However, participants in the latter trial did not have significant sleep disturbance at baseline, which may have affected the results. Perhaps for this reason, meta-analysis of the two trials did not find any significant effect on objective sleep parameters, including night-time total sleep time (mean difference: −49.47; 95% CI: −99.70–0.75; I^2^ = 0%.), sleep efficiency (mean difference: 1.85; 95% CI: −3.99–7.69; I^2^ = 71.3%), and wakefulness after sleep onset (mean difference: −17.55; 95% CI: −44.03–8.92; I^2^ = 58.9%). Both trials assessed the effect of the intervention on cognition but found no significant improvement apart from a modest improvement in one domain of the Delis–Kaplan Executive Function System, which is primarily a test of executive function [141].

##### Music

Two pilot trials with small samples of 33 community dwellers [143] and 20 nursing home residents [144], respectively, analysed the preliminary efficacy of music therapy on sleep in persons with cognitive impairment. Both trials found statistically significant improvements in sleep, with one showing improvement in total sleep time measured by actigraphy [143] and the other showing improvement in subjective sleep quality measured using a visual analogue scale [144], suggesting the need to further assess the effectiveness of music therapy in more definitive trials.

##### Other Non-Pharmacological Interventions

Two trials that examined the effects of cognitive educational sessions found significant improvements in both cognition and sleep. In one of these trials, 44 nursing home residents underwent 30 min sessions of individualised learning therapy, comprising reading and arithmetic exercises, twice per week for 12 weeks, with significant improvements in the MMSE and in both the overall and sleep-domain scores of the NPI [145]. The other trial included 108 community-dwellers and involved eight weekly sessions of a multi-domain cognitive training programme incorporating mnemonic techniques to improve *inter alia* memory, visuo-spatial skills, executive function, and concentration [146]. This latter trial demonstrated significant improvements in subjective sleep quality, using the PSQI, and in cognition, using the Clinical Dementia Rating Scale [147].

Finally, in another trial involving 75 nursing home residents [148], a mindfulness intervention comprising eight weekly sessions of mindfulness practice produced significant improvements in subjective sleep quality using the PSQI, the Athens Insomnia Scale [149], and the Insomnia Severity Index [150]. There were also statistically significant improvements in cognition, using the MMSE and the MoCA, and mood, using the Self-Rating Anxiety Scale [151] and the Perceived Stress Scale [152].

#### 3.6.2. Pharmacological Interventions

##### Melatonin

Eleven trials assessed the effect of melatonin (N-acetyl-5-methoxytryptamine), an endogenous indoleamine that is produced nocturnally by the pineal gland following a circadian rhythm to act as a signal of darkness [153]. Two trials used polysomnography to examine the effects of melatonin on the micro-architecture of sleep, and both found statistically significant decreases in latency to all sleep stages [154,155], with one of these trials additionally recording several significant effects on relative electroencephalographic (EEG) power and coherence [155]. Of the nine remaining trials, six produced significant improvements in sleep [88,156,157,158,159,160], while three did not [126,161,162]. A meta-analysis did not show any significant effect of melatonin on night-time total sleep time (mean difference: 20.60; 95% CI: −10.97–52.17; I^2^ = 1.1%) or sleep efficiency (mean difference: −0.64; 95% CI: −6.55–5.26; I^2^ = 0%). Of the eight trials that examined the effect of melatonin on cognition, three found significant improvement [156,157,160], while five did not [88,158,159,161,162]. While two trials achieved significant improvements in functional ability [156,160], two did not [158,162]. Only one trial found significant improvements in behavioural disturbances [159], while four did not [126,158,160,162].

##### Cholinesterase Inhibitors

In total, twelve trials examined the effect of cholinesterase inhibitors on sleep in people with cognitive impairment. Cholinesterase inhibitors enhance cholinergic transmission at neuronal synapses by inhibiting the enzyme, acetylcholinesterase, that hydrolyses the neurotransmitter, acetylcholine [163]. One trial involved hospital in-patients, but the remaining eleven involved community-dwelling participants. None of the trials resulted in a significant worsening of sleep parameters. In terms of improving sleep, the cholinesterase inhibitor rivastigmine had the most evidence. Of the six trials that assessed the effect of rivastigmine on sleep, three found statistically significant improvements [164,165,166], while the remaining three trials produced insignificant trends towards improvement [167,168,169]. One of these trials found rivastigmine produced a significant reduction in REM sleep behaviour disorder amongst participants with the condition for whom initial therapy with melatonin or clonazepam had proven ineffective [164]. Furthermore, in the three trials that examined the effect of rivastigmine on behaviour, significant improvements were found in two [165,166], while a trend towards improvement was noted in the third trial [168]. Another cholinesterase inhibitor, donepezil, produced more mixed results in the eight trials it was studied in. Only two trials produced significant improvements in subjective sleep quality [170,171]. One of these noted that switching donepezil from night-time to morning administration significantly improved subjective sleep quality and daytime sleepiness [171]. Four trials did not find any significant improvement in sleep parameters [165,167,169,172]. The two remaining trials assessing donepezil used polysomnography and did note significant increases in REM percentage [173,174]. Furthermore, one of these trials involved participants with co-morbid obstructive sleep apnoea and noted a significant improvement in apnoea–hypopnoea index and oxygen saturations on donepezil [174]. Finally, the cholinesterase inhibitor galantamine achieved less favourable results. Of the four trials that assessed galantamine, three found no significant improvement in sleep [165,172,175]. In the only trial in which galantamine produced a significant improvement in sleep, a comparison study, both donepezil and rivastigmine failed to do so [169]. However, this was a non-randomised prospective observational trial with a high risk of bias. A meta-analysis of the effects of cholinesterase inhibitors was not possible.

##### Memantine

Memantine is a low-affinity voltage-dependent uncompetitive antagonist of N-methyl-d-aspartate (NMDA) receptors [176] that works to prevent excitotoxicity caused by the excessive concentration of glutamate that is common in AD [177]. Memantine achieved significant improvements in sleep parameters in two trials [170,178]. One of these involved reducing the apparent incidence of REM sleep behaviour disorder amongst participants with Parkinson’s Disease or Dementia with Lewy Bodies [178]. Memantine produced insignificant trends towards improved sleep in the three remaining trials it was involved in [165,179,180]. Memantine also produced significant improvements in behavioural disturbances in all three trials that examined same [165,170,180]. It was not possible to undertake a meta-analysis of the data relating to the effects of memantine.

##### Trazadone

Trazadone is a serotonin antagonist and re-uptake inhibitor initially approved for the treatment of depression [181]. It also has anti-histamine effects and moderates cortisol suppression in the hypothalamic–pituitary axis, which likely contributes to its off-label utility as a treatment for sleep disturbance [181]. Trazadone was examined in two trials involving community-dwelling participants with moderate levels of dementia. Both found significant improvements in objective sleep measures, with no apparent worsening of cognitive symptoms or daytime sleepiness [182,183]. One of these trials found significant improvement in night-time total sleep time and sleep percentage measured using actigraphy [182]. The other trial found that trazadone produced a significant improvement in subjective sleep quality using the PSQI, and, using polysomnography, trazadone was shown to produce significant increases in sleep efficiency and NREM stage 3 whilst producing significant decreases in wakefulness after sleep onset, arousal index, and NREM stage 1 [183]. In this latter trial, trazadone actually improved cognition, anxiety, and daytime sleepiness. However, the results of this trial may be biased by a disproportionately high number of participants being lost to follow-up due to ‘poor response’ and ‘insomnia deterioration’ [183].

##### Orexin-Receptor Antagonists

Two high quality trials examined the effect of orexin-receptor antagonists on sleep in people with cognitive impairment. Orexin is an excitatory neuropeptide secreted by the hypothalamus that plays a role in maintaining wakefulness [184]. The orexin-receptor antagonist suvorexant achieved statistically significant improvements in objective measurements of total sleep time, sleep efficiency, and wakefulness after sleep onset, with significant improvements also recorded for subjective perceptions of sleep quality [185]. Another orexin-receptor antagonist, lemborexant, produced significant improvements in rest–activity rhythm, with different doses of the drug achieving significant improvements in relative amplitude and in the mean activity counts for the five least active hours [186]. Furthermore, some doses showed a significant reduction in the number of night-time awakenings. The 5 mg dose was the only one to reach statistical significance for each of these sleep outcome measures. Nevertheless, despite these positive results, a meta-analysis did not show any significant improvement in night-time total sleep time (mean difference: 9.10; 95% CI: −47.48–65.68; I^2^ = 39.2%) or sleep efficiency (mean difference: 3.89; 95% CI: −0.84–8.63; I^2^ = 0%).

##### Non-Benzodiazepine Sedative-Hypnotics

One trial looking at eszopiclone found significant improvements in objective and subjective sleep measures, including sleep latency, sleep efficiency, total sleep time, and all domains of the PSQI [187]. Additionally, there were significant improvements in cognition, behavioural disturbances, and functional ability. Another trial looked separately at the effect of zopiclone and zolpidem [188]. Zopiclone produced significant improvements in night-time sleep and behaviour. While no significant effect on daytime sleepiness was reported, it was notable that the total daytime sleep time did increase substantially and that two participants in the zopiclone group left the trial due to ‘severe daytime sedation’. Zolpidem significantly reduced wakefulness after sleep onset and the number of night-time awakenings, but the duration of the main night-time sleep actually decreased by an insignificant amount. Whilst neither zopiclone nor zolpidem had any significant effect on total MMSE score, both resulted in significant deteriorations in other cognitive tests. In another trial with a high risk of bias due to a lack of randomisation in treatment group assignment, zolpidem significantly improved subjective sleep quality, as measured by the PSQI, whilst reducing daytime sleepiness, as measured by the ESS [162]. A meta-analysis of the effects of non-benzodiazepine sedative-hypnotics was not possible.

##### Anti-Psychotics

Amongst a cohort of nursing home residents with advanced dementia, the atypical anti-psychotic risperidone significantly increased night-time sleep whilst decreasing daytime sleep [189]. In a separate trial involving community-dwelling participants with more moderate levels of dementia, risperidone improved subjective sleep quality and reduced daytime sleepiness whilst also significantly reducing behavioural disturbances and the incidence of institutionalisation [162]. Another trial involving hospital in-patients with moderate–severe dementia separately examined the effects of quetiapine and haloperidol [190]. Quetiapine significantly reduced the duration of night-time awakenings. While both quetiapine and haloperidol significantly reduced delusions and agitation, quetiapine also significantly improved depression and anxiety. Haloperidol significantly worsened aberrant motor behaviour and caused extra-pyramidal side-effects in two of eleven participants. Both quetiapine and haloperidol significantly improved word recall, while quetiapine also improved word-list memory and produced a non-significant overall increase in MMSE score.

##### Other Pharmacological Interventions

The only included trial that involved mirtazapine, a noradrenergic and specific serotonergic anti-depressant [191], did not show any significant improvement in night-time sleep or cognition whilst producing a significant unwanted increase in daytime sleep after two weeks of 15 mg per night among community dwellers with moderate dementia [192].

## 4. Discussion

This systematic review and meta-analysis of clinical trials examining pharmacological and non-pharmacological interventions to improve sleep in people with cognitive impairment highlights the variety of interventions that have been trialled in this population. Most of the included trials examined non-pharmacological interventions. BLT was studied in more clinical trials than any other intervention found in this review. The results, however, were mixed, and a meta-analysis did not show any significant improvement in sleep parameters using BLT. Most of the trials examining BLT involved nursing home residents with more advanced dementia. Achieving good sleep can be more challenging in institutions [193]. Furthermore, it is possible that neurodegeneration involving the suprachiasmatic nucleus [194] renders those with advanced dementia more impervious to the effects of BLT. Nevertheless, it must be acknowledged that BLT did not achieve significant improvements in sleep in most of the included trials among community-dwelling participants with less severe cognitive impairment. Results were more promising for physical activity interventions and for the use of CPAP amongst those with co-morbid sleep disordered breathing. In both cases, most of the included trials demonstrated significant improvements in sleep parameters, whilst all of the included trials that measured cognition found significant improvements post-intervention. Other non-pharmacological interventions (such as trans-cranial magnetic stimulation, CBT-I, music therapy, tai chi, and cognitive educational sessions) showed promise in a small number of trials but did not show efficacy in meta-analysis and require further research to clarify their effectiveness in this population.

Among the pharmacological interventions included in this review, melatonin was the most studied. While melatonin produced significant improvements in sleep in eight of the eleven trials it was involved in, our meta-analysis did not demonstrate any statistically significant improvement in sleep. However, all eight trials in which melatonin produced significant improvements in sleep examined lower doses of 6 mg or less, whereas two of the three trials in which melatonin failed to produce any significant improvement in sleep involved higher doses of 6 mg or more. To further illustrate this point, in the one trial that tested two different doses of melatonin, the 2.5 mg dose produced significant improvement in sleep, while the 10 mg dose failed to do so [159]. This finding that lower doses of melatonin may be more effective has been suggested previously [195,196] and warrants further investigation. Among the cholinesterase inhibitors, rivastigmine appears to have the best evidence for improving sleep, including one trial in which it produced a significant reduction in intractable REM sleep behaviour disorder [164]. While the evidence for donepezil is less compelling, it is notable that switching the time of administration to the morning improved subjective sleep quality in one trial [171]. Furthermore, in another trial [174], donepezil produced a significant improvement in the apnoea–hypopnoea index and oxygen saturation levels of people with co-morbid obstructive sleep apnoea, suggesting that donepezil may be an option to ameliorate this condition in people with cognitive impairment who fail to tolerate CPAP. Galantamine did not have any positive effect on sleep. Memantine improved sleep in all of the trials found in this review, but most of these did not achieve statistical significance. However, it did produce significant improvements in all trials that assessed behavioural disturbance as an outcome measure. Trazadone showed promise by improving sleep without adversely affecting daytime sleepiness in two trials involving community-dwelling participants with moderate dementia [182,183]. Orexin-receptor antagonists demonstrated significant improvements in sleep in two high quality trials [185,186]. Given the implication of the orexinergic system in the pathophysiology of Alzheimer’s Disease [197], there is potential for orexin-receptor antagonists to emerge as important therapeutic options [198]. It must be acknowledged, however, that orexin-receptor antagonists did not produce any significant improvement in cognition in either of the two aforementioned trials. The non-benzodiazepine sedative–hypnotic eszopiclone produced significant improvements in sleep and cognition in one trial involving hospital in-patients [187]. The evidence for zopiclone and zolpidem is less compelling. Whilst zopiclone produced significant improvements in sleep in one trial, there was disconcerting evidence of ‘hangover’ daytime sleepiness [188]. Zolpidem failed to improve the duration of night-time sleep in the same trial, and, although it did significantly improve subjective sleep quality in another trial, there were concerns regarding the methodological quality of the latter trial due to a lack of randomisation in treatment group assignment [162]. Regarding the use of anti-psychotic medications in people with moderate–severe dementia, there is some poor-quality evidence that risperidone and quetiapine may be beneficial for sleep, but, again, further research is required given the risk of adverse events, especially cardiovascular risk and extrapyramidal side effects, with a prolonged use of these medications in this population [199].

Overall, many of the trials included in this review were characterised by poor methodological quality and a high risk of bias. This is an issue that has been highlighted and discussed previously [31]. Attrition bias was the most common reason why trials included in this review were adjudged to be at high risk of bias. Whilst it is understandable, given the nature of the populations involved, that there would be relatively high rates of attrition in the included trials, the consequent risk of bias could be significantly ameliorated by an adequate description of the reasons for attrition.

There was wide heterogeneity in the methods employed to measure sleep in the included trials. Subjective measures were by far the most commonly used, which is perhaps unsurprising given their ease of administration and the relatively low burden imposed on participants [200]. Moreover, improving the subjective experience of sleep is an important treatment goal in itself. However, subjective measures of sleep are prone to recall bias, an issue that is particularly relevant for people with cognitive impairment [31]. Several studies have demonstrated discrepancies between subjective and objective measurements of sleep in people with cognitive impairment [201,202]. Proxy observer reports are also prone to similar sleep misperception [23,203], though this is perhaps less of an issue for trained observers [204] who have demonstrated excellent inter-rater reliability in some clinical trials [95].

Polysomnography is the recognised gold standard method of measuring sleep [205]. Given emerging evidence about the changes in the macro and micro-architecture of sleep associated with cognitive impairment [3,4] and the longitudinal implication of these changes in cognitive decline [206], relatively few trials (*n* = 14) used polysomnography to measure sleep. Further evidence is required to definitively clarify the effect that changes in these sleep parameters have on cognitive decline [206]. Whilst polysomnography can provide a precise picture of sleep architecture, monitoring is rather invasive and expensive and generally spans shorter periods of time, thus limiting the ability to assess patterns of circadian rhythm [207]. Some researchers have indeed questioned whether polysomnography can be regarded as the gold standard method of measuring sleep in people with dementia [208]. The abundance of diffuse slow activity during wakefulness in people with advanced dementia makes it difficult to distinguish between sleep and wakefulness [209]. Additionally, polysomnography is not recommended for people with dementia because it relies on participants’ understanding of the method [31]. The so-called ‘first night effects’ [210], whereby participants experience worse sleep due to the unfamiliar sleeping environment in the sleep laboratory and the discomfort associated with wearing the measurement equipment are likely to be particularly troublesome in this population [31]. Perhaps some of these obstacles can be overcome in future with the emergence of commercially available home-based EEG recording devices [211], but, so far, it appears that the adoption of these devices has been slow [29].

Actigraphy has gained widespread acceptance as a surrogate objective measure of sleep [206,212]. Actigraphic measurements of sleep parameters have been shown to correlate closely with polysomnography [212], including in older patients [213]. By allowing for continuous recording over multiple days, actigraphy offers a broader assessment of sleep and additional insights into circadian patterns [206], all of which can be obtained from the comfort of the participants’ home environment [31]. However, actigraphy may over-estimate sleep due to difficulties distinguishing motionless wakefulness from sleep [214]. Furthermore, whilst actigraphy has been shown to be acceptable for use in people with dementia [215,216], there are concerns that its accuracy tends to decline in more advanced cognitive impairment [217].

Each of the different sleep measurement methods discussed herein has its own advantages and disadvantages, capturing different aspects of sleep quality [217]. It is difficult, therefore, to argue with confidence regarding the best method of measuring sleep in people with cognitive impairment. Perhaps the best solution is to incorporate both subjective and objective measures [30] in a multi-modal approach to assessing sleep–wake activity [217]. In addition to the foregoing considerations regarding the most appropriate method to measure sleep in clinical trials, it is also important to ensure that there is consistency in the specific outcome measures reported. This review reveals marked heterogeneity, with 287 discreet sleep outcome measures used, 205 of which were used in only one of the included trials. No single outcome measure was used in over half of the included trials. This heterogeneity severely restricts opportunities for data synthesis, which is particularly problematic given that most of the trials included in this review involved small sample sizes.

### Limitations

In addition to heterogeneity between studies limiting the ability to perform meta-analysis, this study has other limitations. While comprehensive, some studies may not have been included. There was a significant number of unreported data compounding this. For example, information on the timing of assessing outcome measures was variable, and it is likely that this was another source of heterogeneity. Additional research is therefore required to develop a core outcome set (COS) to standardise reporting including specifying the timing of assessments and the duration of monitoring. Another limitation is that grey literature was not searched due to the challenges inherent in searching for this and a lack of availability of the additional resources this would entail. While it is acknowledged that excluding grey literature in this manner may compromise the reliability of the findings of this review, it is also recognised that data contained in grey literature is of variable quality and often does not meaningfully impact the results of meta-analysis. Similarly, excluding articles not written in English may have introduced selection bias, particularly as studies pertaining to certain intervention modalities (such as traditional or herbal interventions) may more likely be reported in other languages.

Given the challenges inherent in conducting research among people with cognitive impairment [218], this review serves to underpin the development of a COS for clinical trials of interventions to improve sleep in people with cognitive impairment—the Sleep in Cognitive Impairment Core Outcome Set (SCICOS) study [40]. The SCICOS will help to ensure that future clinical trials contribute to a reliable and coherent body of evidence upon which clinical practice decisions can be based with ever-growing confidence.

## 5. Conclusions

This systematic review and meta-analysis of pharmacological and non-pharmacological interventions to improve sleep in people with cognitive impairment illustrates that, while many different therapies and interventions have been trialled, most do not as yet have sufficiently robust evidence upon which to confidently base clinical practice decisions. Regarding non-pharmacological interventions, there is good evidence for physical activity and for CPAP amongst those with sleep disordered breathing. Trazadone and the orexin-receptor antagonists are the pharmacological interventions with the best evidence to support their effectiveness in treating sleep disturbances in people with cognitive impairment. Further evidence is required, however, before confident recommendations could be made regarding their use in clinical practice. If prescribing a cholinesterase inhibitor for cognitive impairment, there is evidence to suggest that rivastigmine would be the most effective option to help treat co-morbid sleep disturbance as an ancillary benefit.

Given the increasing prevalence of dementia and the implication of sleep disturbance both in its pathophysiology and in its symptomatology, understanding how best to treat it is an emerging and important public health issue. Hence, there is an urgent need for further research to clarify the most effective interventions to improve sleep among this population [219]. However, many of the clinical trials conducted in this field are limited by concerns regarding methodological quality, with wide heterogeneity in the outcome measures reported further limiting opportunities for data synthesis. In addition to updating the evidence for both pharmacological and non-pharmacological interventions in this population, this review will serve to underpin the development of a core outcome set that will help ensure that future clinical trials contribute to a more reliable and coherent evidence base, to improve the lives of people living with cognitive impairment and their families [40].

## Figures and Tables

**Table 1:** Summary of eligibility criteria for inclusion in this systematic review.

Inclusion Criteria	Exclusion Criteria
Original peer reviewed articles Written in the English language Clinical trials of interventions to improve sleep involving: ○ participants: ▪ aged ≥ 18 years ▪ with MCI or dementia ○ contemporaneous comparison of the intervention with at least one other control group ○ at least one primary or secondary outcome relating to sleep	Participants who are: ○ post-stroke patients ○ post-traumatic brain injury patients ○ in-patients in intensive care units ○ oncology patients Grey literature

## Data Availability

The original contributions presented in this study are included in the article/Supplementary Materials. Further inquiries can be directed to the corresponding author(s).

## Supplementary Materials

The following supporting information can be downloaded at: https://www.mdpi.com/article/10.3390/ijerph22060956/s1. Table S1: Search strategies; Table S2: Characteristics of the included trials [46,67,68,69,70,71,72,73,74,75,76,77,78,79,80,81,82,83,84,85,86,87,88,89,92,93,94,95,96,97,98,99,100,101,102,103,104,105,106,107,108,109,110,111,112,113,114,115,116,117,118,119,120,121,122,123,124,125,126,127,128,129,131,132,133,134,135,136,137,138,139,140,141,142,143,144,145,146,148,154,155,156,157,158,159,160,161,162,164,165,166,167,168,169,170,171,172,173,174,175,178,179,180,182,183,185,186,187,188,189,190,192,220,221,222,223,224,225,226,227,228,229,230,231,232,233,234,235,236,237,238,239,240,241,242,243,244,245,246,247,248,249,250,251]; Table S3: Quality and risk of bias assessment; Table S4: Sleep Outcome Measures; Figure S1a–i: Meta-analyses; Figure S2: The sleep outcome measures reported in only one trial.
