# Supplementary material for: Pharmacological and Non-Pharmacological Interventions to Improve Sleep in People with Cognitive Impairment: A Systematic Review and Meta-Analysis
Source: Int J Environ Res Public Health. 2025 Jun 18;22(6):956. doi: 10.3390/ijerph22060956 (PMC12192850; doi:10.3390/ijerph22060956)
Supplement: Supplementary file 1 [file ijerph-22-00956-s001.zip › Figures_S1_Meta-analyses.pdf]

Bright Light Therapy

Study (cluster)	Intervention	Intervention Total	Intervention Mean	Intervention SD	Control Total	Control Mean	Control SD	MD	95%-CI	Weight
Friedman et al. 2012	Bright Light Therapy	31	393.00	127.00	23	420.00	125.00	-27.00	[-94.88; 40.88]	13.5%
Sloane et al. 2015	Bright Light Therapy	15	246.30	77.90	16	248.20	73.30	-1.90	[-55.23; 51.43]	21.0%
Dowling et al. 2005	Morning Light	29	479.00	114.00	17	512.00	120.00	-33.00	[-103.54; 37.54]	12.2%
Dowling et al. 2005	Evening Light	24	522.00	98.00	17	512.00	120.00	10.00	[-59.22; 79.22]	12.6%
Burns et al. 2009	Bright Light Therapy	20	530.00	60.00	22	501.00	61.00	29.00	[-7.62; 65.62]	39.7%
Fontana Gasio et al. 2002	Dawn-Dusk Simulation Therapy	9	576.00	388.00	4	433.00	90.00	143.00	[-125.39; 411.39]	0.9%
**Random effects model**		**128**			**99**			**6.02**	**[-27.97; 40.02]**	**100.0%**
**Prediction interval**									**[-35.92; 47.97]**	

Heterogeneity: $I^2 = 0.0\%$ [0.0%; 74.6%], $\tau^2 = 91.41$, $p = 0.455$

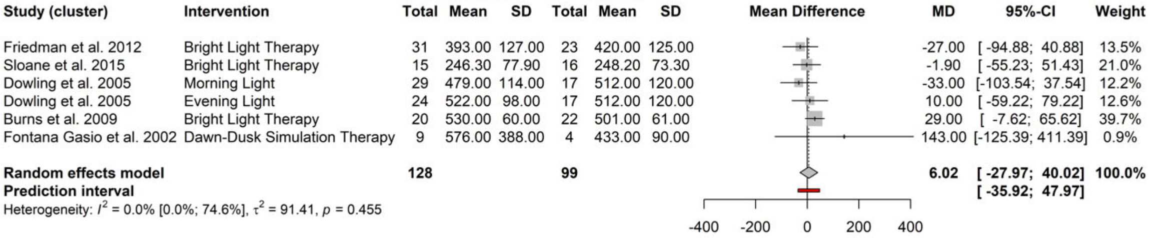

Figure S1a. Meta-analysis of the effect of Bright Light Therapy on Night-time Total Sleep Time.

Study (cluster)	Intervention	Intervention Total	Intervention Mean	Intervention SD	Control Total	Control Mean	Control SD	MD	95%-CI	Weight
Cremascoli et al. 2022	Bright Light Therapy	8	78.74	5.57	5	83.77	10.40	-5.03	[-14.93; 4.87]	20.9%
Friedman et al. 2012	Bright Light Therapy	31	71.70	21.20	23	75.80	20.80	-4.10	[-15.41; 7.21]	19.1%
Sloane et al. 2015	Bright Light Therapy	15	68.40	21.60	16	68.90	20.30	-0.50	[-15.28; 14.28]	15.3%
Dowling et al. 2005	Morning Light	29	66.64	15.85	17	71.14	16.78	-4.50	[-14.34; 5.34]	12.1%
Dowling et al. 2005	Evening Light	24	72.68	13.65	17	71.14	16.78	1.54	[-8.13; 11.21]	12.5%
Fontana Gasio et al. 2002	Dawn-Dusk Simulation Therapy	9	80.40	2.10	4	62.60	10.60	17.80	[7.32; 28.28]	20.1%
**Random effects model**		**116**			**82**			**1.32**	**[-9.60; 12.25]**	**100.0%**
**Prediction interval**									**[-21.55; 24.20]**	

Heterogeneity: $I^2 = 62.5\%$ [8.8%; 84.6%], $\tau^2 = 61.14$, $p = 0.020$

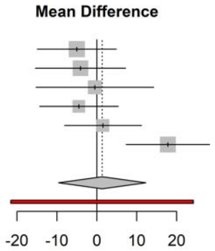

Figure S1b. Meta-analysis of the effect of Bright Light Therapy on Sleep Efficiency.

Cognitive Behavioural Therapy for Insomnia

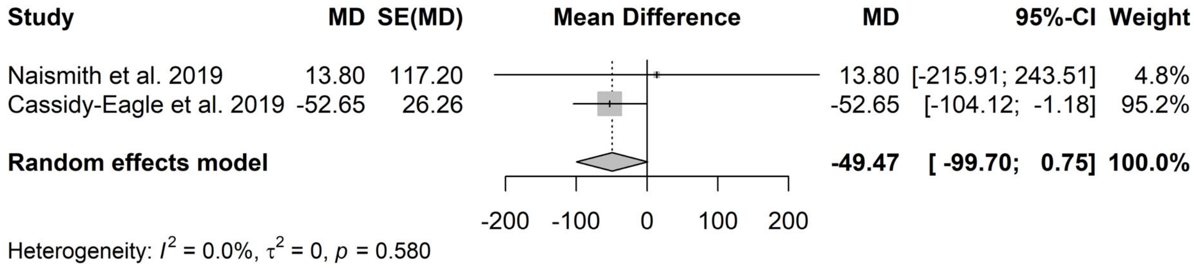

Figure S1c. Meta-analysis of the effect of Cognitive Behavioural Therapy for Insomnia on Night-time Total Sleep Time.

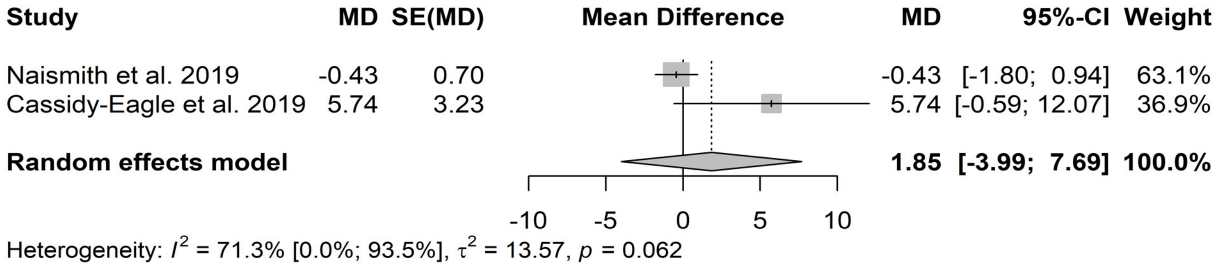

Figure S1d. Meta-analysis of the effect of Cognitive Behavioural Therapy for Insomnia on Sleep Efficiency.

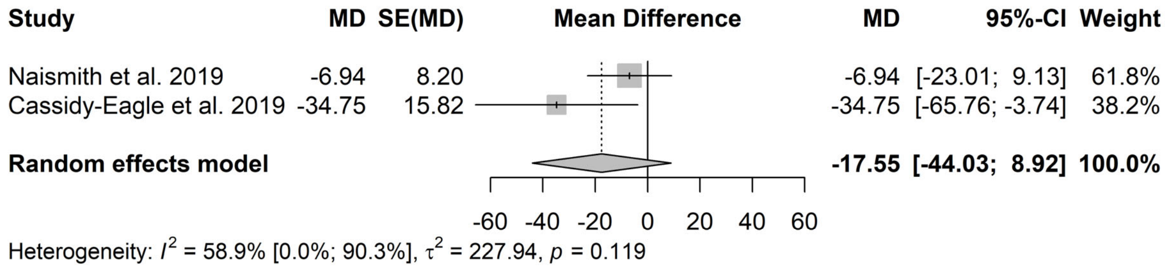

Figure S1e. Meta-analysis of the effect of Cognitive Behavioural Therapy for Insomnia on Wakefulness After Sleep Onset.

Melatonin

Study (cluster)	Intervention	Intervention Total	Intervention Mean	Intervention SD	Control Total	Control Mean	Control SD	MD	95%-CI	Weight
Gehrman et al. 2009	Melatonin 10mg	24	450.00	120.00	17	474.00	138.00	-24.00	[-105.29; 57.29]	5.7%
Singer et al. 2003	Melatonin 2.5mg	54	375.70	76.20	47	349.40	88.40	26.30	[-6.13; 58.73]	35.9%
Singer et al. 2003	Melatonin 10mg	50	357.80	70.60	47	349.40	88.40	8.40	[-23.56; 40.36]	37.0%
Asayama et al. 2003	Melatonin 3mg	11	427.10	29.30	9	383.00	58.70	44.10	[2.02; 86.18]	21.3%
**Random effects model**		**139**			**120**			**20.60**	**[-10.97; 52.17]**	**100.0%**
**Prediction interval**									**[-10.97; 52.17]**	

Heterogeneity: I^2 = 1.1% [0.0%; 84.9%], τ^2 < 0.01, p = 0.387

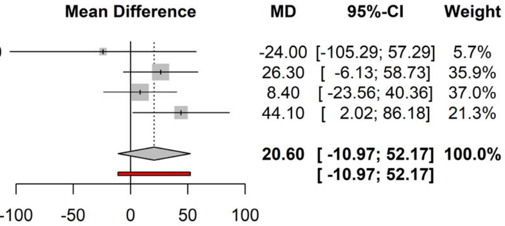

Figure S1f. Meta-analysis of the effect of Melatonin on Night-time Total Sleep Time.

Study (cluster)	Intervention	Intervention Total	Intervention Mean	Intervention SD	Control Total	Control Mean	Control SD	MD	95%-CI	Weight
Gehrman et al. 2009	Melatonin 10mg	24	78.60	14.60	17	80.70	18.10	-2.10	[-12.50; 8.30]	6.7%
Singer et al. 2003	Melatonin 2.5mg	54	69.00	11.00	47	69.00	10.00	0.00	[-4.10; 4.10]	43.1%
Singer et al. 2003	Melatonin 10mg	50	68.00	9.00	47	69.00	10.00	-1.00	[-4.79; 2.79]	50.2%
**Random effects model**		**128**			**111**			**-0.64**	**[-6.55; 5.26]**	**100.0%**
**Prediction interval**									**[-6.55; 5.26]**	

Heterogeneity: I^2 = 0.0% [0.0%; 89.6%], τ^2 < 0.01, p = 0.903

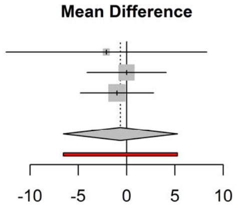

Figure S1g. Meta-analysis of the effect of Melatonin on Sleep Efficiency.

Orexin-Receptor Antagonists

Study (cluster)	Intervention	Intervention Total	Intervention Mean	Intervention SD	Control Total	Control Mean	Control SD	MD	95%-CI	Weight
Moline et al. 2021	Lemburexant 2.5mg	12	395.56	67.27	12	421.76	57.18	-26.20	[-76.15; 23.75]	13.2%
Moline et al. 2021	Lemburexant 5mg	13	419.26	83.45	12	421.76	57.18	-2.50	[-58.22; 53.22]	10.6%
Moline et al. 2021	Lemburexant 10mg	13	412.75	89.53	12	421.76	57.18	-9.01	[-67.45; 49.43]	9.6%
Moline et al. 2021	Lemburexant 15mg	12	412.50	64.24	12	421.76	57.18	-9.26	[-57.92; 39.40]	13.9%
Herring et al. 2020	Suvorexant 20mg	135	349.40	71.90	139	321.00	85.20	28.40	[9.75; 47.05]	52.7%
**Random effects model**		**185**			**187**			**9.10**	**[-47.48; 65.68]**	**100.0%**
**Prediction interval**									**[-83.50; 101.70]**	

Heterogeneity: I^2 = 39.2% [0.0%; 77.5%], τ^2 = 697.19, p = 0.160

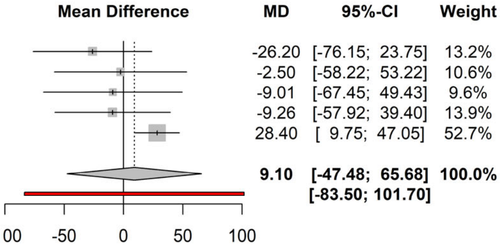

Figure S1h. Meta-analysis of the effect of Orexin-Receptor Antagonists on Night-time Total Sleep Time.

Study (cluster)	Intervention	Intervention Total	Intervention Mean	Intervention SD	Control Total	Control Mean	Control SD	MD	95%-CI	Weight
Moline et al. 2021	Lemburexant 2.5mg	12	79.85	6.89	12	75.09	7.69	4.76	[-1.08; 10.60]	16.4%
Moline et al. 2021	Lemburexant 5mg	13	78.19	7.92	12	75.09	7.69	3.10	[-3.02; 9.22]	14.9%
Moline et al. 2021	Lemburexant 10mg	13	74.32	9.26	12	75.09	7.69	-0.77	[-7.42; 5.88]	12.7%
Moline et al. 2021	Lemburexant 15mg	12	76.54	9.65	12	75.09	7.69	1.45	[-5.53; 8.43]	11.5%
Herring et al. 2020	Suvorexant 20mg	135	72.80	14.90	139	67.00	17.80	5.80	[1.92; 9.68]	44.5%
**Random effects model**		**185**			**187**			**3.89**	**[-0.84; 8.63]**	**100.0%**
**Prediction interval**									**[-2.63; 10.42]**	

Heterogeneity: I^2 = 0.0% [0.0%; 79.2%], τ^2 = 2.62, p = 0.490

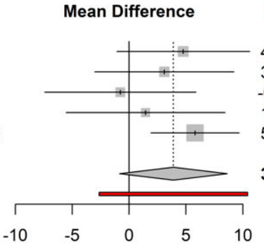

Figure S1i. Meta-analysis of the effect of Orexin-Receptor Antagonists on Sleep Efficiency.
