# Supplementary material for: Pharmacological and Non-Pharmacological Interventions to Improve Sleep in People with Cognitive Impairment: A Systematic Review and Meta-Analysis
Source: Int J Environ Res Public Health. 2025 Jun 18;22(6):956. doi: 10.3390/ijerph22060956 (PMC12192850; doi:10.3390/ijerph22060956)
Supplement: Supplementary file 1 [file ijerph-22-00956-s001.zip › Table_S1_Search Strategies.pdf]

Supplementary Materials

Table S1. Search strategies

Medline (Ovid)

1	sleep.sh,tw.
2	insomnia.sh,tw.
3	circadian.sh,tw.
4	1 or 2 or 3
5	dement*.sh,tw.
6	"alzheimer*".sh,tw.
7	"cogniti*".sh,tw.
8	"neurocogniti*".sh,tw.
9	"nursing home*".sh,tw.
10	long term care.sh,tw.
11	"residential facilit*".sh,tw.
12	residential care.sh,tw.
13	5 or 6 or 7 or 8 or 9 or 10 or 11 or 12
14	(Randomized Controlled Trial or Controlled Clinical Trial or Pragmatic Clinical Trial or Clinical Study or Adaptive Clinical Trial or Equivalence Trial).pt.
15	(Clinical Trial or Clinical Trial, Phase I or Clinical Trial, Phase II or Clinical Trial, Phase III or Clinical Trial, Phase IV or Clinical Trial Protocol).pt.
16	Multicenter Study.pt.
17	Clinical Studies as Topic/
18	exp Clinical Trial/ or exp Clinical Trials as Topic/ or Clinical Trial Protocol/ or Clinical Trial Protocols as Topic/ or exp "Clinical Trial (topic)"/
19	Multicenter Study/ or Multicenter Studies as Topic/ or "Multicenter Study (topic)"/
20	Randomization/
21	Random Allocation/
22	Double-Blind Method/
23	Double Blind Procedure/
24	Double-Blind Studies/
25	Single-Blind Method/
26	Single Blind Procedure/
27	Single-Blind Studies/
28	Placebos/
29	Placebo/
30	Control Groups/
31	Control Group/
32	Cross-Over Studies/ or Crossover Procedure/
33	(random* or sham or placebo*).ti,ab,hw,kf.
34	((singl* or doubl*) adj (blind* or dumm* or mask*)).ti,ab,hw,kf.
35	((tripl* or trebl*) adj (blind* or dumm* or mask*)).ti,ab,hw,kf.
36	(control* adj3 (study or studies or trial* or group*)).ti,ab,hw,kf.
37	(clinical adj3 (study or studies or trial*)).ti,ab,hw,kf.

38	(Nonrandom* or non random* or non-random* or quasi-random* or quasirandom*).ti,ab,hw,kf.
39	(phase adj3 (study or studies or trial*)).ti,ab,hw,kf.
40	((crossover or cross-over) adj3 (study or studies or trial*)).ti,ab,hw,kf.
41	((multicent* or multi-cent*) adj3 (study or studies or trial*)).ti,ab,hw,kf.
42	allocated.ti,ab,hw.
43	((open label or open-label) adj5 (study or studies or trial*)).ti,ab,hw,kf.
44	((equivalence or superiority or non-inferiority or noninferiority) adj3 (study or studies or trial*)).ti,ab,hw,kf.
45	(pragmatic study or pragmatic studies).ti,ab,hw,kf.
46	((pragmatic or practical) adj3 trial*).ti,ab,hw,kf.
47	((quasiexperimental or quasi-experimental) adj3 (study or studies or trial*)).ti,ab,hw,kf.
48	trial.ti,kf.
49	14 or 15 or 16 or 17 or 18 or 19 or 20 or 21 or 22 or 23 or 24 or 25 or 26 or 27 or 28 or 29 or 30 or 31 or 32 or 33 or 34 or 35 or 36 or 37 or 38 or 39 or 40 or 41 or 42 or 43 or 44 or 45 or 46 or 47 or 48
50	exp animals/
51	exp animal experimentation/
52	exp models animal/
53	exp animal experiment/
54	nonhuman/
55	exp vertebrate/
56	50 or 51 or 52 or 53 or 54 or 55
57	exp humans/
58	56 not 57
59	49 not 58
60	4 and 13 and 59

<u>The Cochrane Library CENTRAL database</u>

1	sleep.sh,tw.
2	insomnia.sh,tw.
3	circadian.sh,tw.
4	1 or 2 or 3
5	dement*.sh,tw.
6	"alzheimer*".sh,tw.
7	"cogniti*".sh,tw.
8	"neurocogniti*".sh,tw.
9	"nursing home*".sh,tw.
10	long term care.sh,tw.
11	"residential facilit*".sh,tw.
12	residential care.sh,tw.
13	5 or 6 or 7 or 8 or 9 or 10 or 11 or 12
14	4 and 13

<u>CINAHL (EBSCO)</u>

1	AB sleep
2	MJ sleep
3	AB insomnia
4	MJ insomnia
5	AB circadian
6	MJ circadian
7	1 or 2 or 3 or 4 or 5 or 6
8	AB dement*
9	MJ dement*
10	AB alzheimer*
11	MJ alzheimer*
12	AB cogniti*
13	MJ cogniti*
14	AB neurocogniti*
15	MJ neurocogniti*
16	AB nursing home*
17	MJ nursing home*
18	AB residential care
19	MJ residential care
20	AB residential facilit*
21	MJ residential facilit*
22	AB long term care
23	MJ long term care
24	8 or 9 or 10 or 11 or 12 or 13 or 14 or 15 or 16 or 17 or 18 or 19 or 20 or 21 or 22 or 23 or 24
25	((MH "Experimental Studies+") OR (MH "Multicenter Studies") OR (MH "Random Sample+") OR (MH "Placebos") OR (MH "Control (Research)+") OR (MH "Crossover Design") OR ((TI random* OR AB random*) OR (TI sham OR AB sham) OR (TI placebo* OR AB placebo*)) OR (((TI singl* OR AB singl*) OR (TI doubl* OR AB doubl*)) W1 ((TI blind* OR AB blind*) OR (TI dumm* OR AB dumm*) OR (TI mask* OR AB mask*))) OR (((TI tripl* OR AB tripl*) OR (TI trebl* OR AB trebl*)) W1 ((TI blind* OR AB blind*) OR (TI dumm* OR AB dumm*) OR (TI mask* OR AB mask*))) OR ((TI control* OR AB control*) N3 ((TI study OR AB study) OR (TI studies OR AB studies) OR (TI trial* OR AB trial*) OR (TI group* OR AB group*))) OR ((TI clinical OR AB clinical) N3 ((TI study OR AB study) OR (TI studies OR AB studies) OR (TI trial* OR AB trial*))) OR ((TI Nonrandom* OR AB Nonrandom*) OR (TI "non random*" OR AB "non random*") OR (TI "non-random*" OR AB "non-random*") OR (TI "quasi-random*" OR AB "quasi-random*") OR (TI quasirandom* OR AB quasirandom*)) OR ((TI phase OR AB phase) N3 ((TI study OR AB study) OR (TI studies OR AB studies) OR (TI trial* OR AB trial*))) OR (((TI crossover OR AB crossover) OR (TI "cross-over" OR AB "cross-over")) N3 ((TI study OR AB study) OR (TI studies OR AB studies) OR (TI trial* OR AB trial*))) OR (((TI multicent* OR AB multicent*) OR (TI "multi-cent*" OR AB "multi-cent*")) N3 ((TI study OR AB study) OR (TI studies OR AB studies) OR (TI trial* OR AB trial*))) OR (TI allocated OR AB allocated) OR (((TI "open label" OR AB "open label") OR (TI "open-label" OR AB "open-label")) N5 ((TI study OR AB study) OR (TI studies OR AB studies) OR (TI trial* OR AB trial*))) OR (((TI equivalence OR AB equivalence) OR (TI superiority OR AB superiority) OR (TI "non-inferiority" OR AB "non-inferiority") OR (TI noninferiority OR AB noninferiority)) N3 ((TI study OR AB study) OR (TI studies OR AB studies) OR (TI trial* OR AB trial*))) OR ((TI "pragmatic study" OR AB "pragmatic study") OR (TI "pragmatic studies" OR AB "pragmatic studies")) OR (((TI pragmatic OR AB pragmatic) OR (TI practical OR AB practical)) N3 (TI trial* OR AB trial*))

	OR (((TI quasiexperimental OR AB quasiexperimental) OR (TI "quasi-experimental" OR AB "quasi-experimental")) N3 ((TI study OR AB study) OR (TI studies OR AB studies) OR (TI trial* OR AB trial*))) OR (TI trial))
26	7 and 24 and 25

PsycINFO (EBSCO)

1	AB sleep
2	MA sleep
3	AB insomnia
4	MA insomnia
5	AB circadian
6	MA circadian
7	1 or 2 or 3 or 4 or 5 or 6
8	AB dement*
9	MA dement*
10	AB alzheimer*
11	MA alzheimer*
12	AB cogniti*
13	MA cogniti*
14	AB neurocogniti*
15	MA neurocogniti*
16	AB nursing home*
17	MA nursing home*
18	AB residential care
19	MA residential care
20	AB residential facilit*
21	MA residential facilit*
22	AB long term care
23	MA long term care
24	8 or 9 or 10 or 11 or 12 or 13 or 14 or 15 or 16 or 17 or 18 or 19 or 20 or 21 or 22 or 23 or 24
25	((MH "Experimental Studies+") OR (MH "Multicenter Studies") OR (MH "Random Sample+") OR (MH "Placebos") OR (MH "Control (Research)+") OR (MH "Crossover Design") OR ((TI random* OR AB random*) OR (TI sham OR AB sham) OR (TI placebo* OR AB placebo*)) OR (((TI singl* OR AB singl*) OR (TI doubl* OR AB doubl*)) W1 ((TI blind* OR AB blind*) OR (TI dumm* OR AB dumm*) OR (TI mask* OR AB mask*))) OR (((TI tripl* OR AB tripl*) OR (TI trebl* OR AB trebl*)) W1 ((TI blind* OR AB blind*) OR (TI dumm* OR AB dumm*) OR (TI mask* OR AB mask*))) OR ((TI control* OR AB control*) N3 ((TI study OR AB study) OR (TI studies OR AB studies) OR (TI trial* OR AB trial*) OR (TI group* OR AB group*))) OR ((TI clinical OR AB clinical) N3 ((TI study OR AB study) OR (TI studies OR AB studies) OR (TI trial* OR AB trial*))) OR ((TI Nonrandom* OR AB Nonrandom*) OR (TI "non random*" OR AB "non random*") OR (TI "non-random*" OR AB "non-random*") OR (TI "quasi-random*" OR AB "quasi-random*") OR (TI quasirandom* OR AB quasirandom*)) OR ((TI phase OR AB phase) N3 ((TI study OR AB study) OR (TI studies OR AB studies) OR (TI trial* OR AB trial*))) OR (((TI crossover OR AB crossover) OR (TI "cross-over" OR AB "cross-over")) N3 ((TI study OR AB study) OR (TI studies OR AB studies) OR (TI trial* OR AB trial*))) OR (((TI multicent* OR AB multicent*) OR (TI "multi-cent*" OR AB "multi-cent*")) N3 ((TI study OR AB study) OR (TI studies OR AB studies) OR (TI trial* OR AB trial*))) OR (TI allocated OR AB allocated) OR (((TI "open label" OR AB "open label") OR (TI "open-label" OR AB "open-label")) N5 ((TI study OR AB

	study) OR (TI studies OR AB studies) OR (TI trial* OR AB trial*))) OR (((TI equivalence OR AB equivalence) OR (TI superiority OR AB superiority) OR (TI "non-inferiority" OR AB "non-inferiority") OR (TI noninferiority OR AB noninferiority)) N3 ((TI study OR AB study) OR (TI studies OR AB studies) OR (TI trial* OR AB trial*))) OR ((TI "pragmatic study" OR AB "pragmatic study") OR (TI "pragmatic studies" OR AB "pragmatic studies")) OR (((TI pragmatic OR AB pragmatic) OR (TI practical OR AB practical)) N3 (TI trial* OR AB trial*)) OR (((TI quasiexperimental OR AB quasiexperimental) OR (TI "quasi-experimental" OR AB "quasi-experimental")) N3 ((TI study OR AB study) OR (TI studies OR AB studies) OR (TI trial* OR AB trial*))) OR (TI trial))
26	7 and 24 and 25
