# Supplementary material for: Pharmacological and Non-Pharmacological Interventions to Improve Sleep in People with Cognitive Impairment: A Systematic Review and Meta-Analysis
Source: Int J Environ Res Public Health. 2025 Jun 18;22(6):956. doi: 10.3390/ijerph22060956 (PMC12192850; doi:10.3390/ijerph22060956)
Supplement: Supplementary file 1 [file ijerph-22-00956-s001.zip › Table_S2_Characteristics of the included trials.pdf]

First Author	Publication Year	Country	Setting	Number of Participants randomised	Participants Age [mean(SD)]	Baseline Cognition - MMSE unless otherwise stated [mean(SD)]	Trial Design	Intervention	Intervention Duration	Control	Sleep Measurement Tool(s)	Sleep Outcome Measure(s)	Result Summary
Alessi [46]	1995	USA	NH	65	IG: 84.4(7.2); CG: 85.1(7.6)	IG: 12.3(8.1); CG: 13.8(7.9)	RCT	Physical Activity - Functional Intervention Training (FIT)	9 weeks	Row/Walk/Wheel (RWW)	Wrist actigraphy; Observer report;	NTST; Night % sleep; Average duration of sleep episodes (minutes); Peak duration of sleep episodes (minutes); Daytime Sleep (% observations);	No significant improvement in sleep parameters.
Alessi [92]	1999	USA	NH	29	IG: 88.6(10.4) CG: 88.3(5.7)	IG: 13.6(8.5); CG: 13.1(8.1)	RCT	Daytime: Physical activity (FIT); Nighttime: Reduced Noise, Light and Nursing Intervention	14 weeks	Nighttime Intervention alone	Wrist actigraphy; Observer report;	Night % sleep; Max duration of sleep episodes (minutes); Average duration of sleep episodes (minutes); Daytime in bed (% observations); Daytime sleep (% observations); Daytime agitation (% observations);	Significant increase in night-time sleep percentage, with significant reductions in daytime agitation and time in bed.
Alessi [95]	2005	USA	NH	118	IG 87.8(7.8) CG 85.9(10.1)	IG 11.9(9.2) CG 10.6(10.0)	RCT	Multi-dimensional:<br>Day: 1. Sunlight exposure 2. Reduced daytime in bed 3. Increased daytime exercise.<br>Night: 1. Reduced light, 2. Reduced noise, 3. Reduced nursing intervention	5 days	Usual care	Wrist actigraphy; Observer report;	NTST; Night % sleep; NNA; Mean duration of nighttime awakenings; Daytime Sleep (% observations); DTIB (% observations)	Significant decrease in daytime sleep and daytime time in bed. No significant effect on night-time sleep apart from a modest reduction in mean duration of night-time awakenings. Significantly increased physical activity and participation in social activities and conversation.
Ancoli-Israel [68]	2002	USA	NH	77	85.7(7.3)	12.8(8.8)	RCT	4 groups:<br>Morning Bright Light; Evening Bright Light; Evening Dim Red Light; Daytime Sleep Restriction;	10 days	Daytime Sleep Restriction, Evening Dim Red Light.	Wrist actigraphy	Amplitude; Mesor; Acrophase; F value;	No improvement in night-time sleep or daytime alertness. Morning bright light did significantly delay the peak of the activity rhythm (acrophase) and increase the mean activity level (MESOR).

Ancoli-Israel [86]	2003	USA	NH	92	82.3(7.6)	5.7(5.6)	RCT	3 Groups: Morning Bright Light; Morning Dim Red Light; Evening Bright Light;	10 days	Morning Dim Red Light	Wrist actigraphy	NTST; Night % sleep; Night % awake; Night WASO; DTST; Day % sleep; Day % awake; Day WASO;NNA; Duration of nighttime awakenings. Number of daytime naps; Duration of daytime naps; Length of time between daytime naps. Circadian Activity - F statistic;	Both morning bright light and evening bright light led to longer maximum sleep bout durations at night without improving overall total sleep time. Evening bright light improved rhythmicity, as measured by the F statistic.
Ancoli-Israel [172]	2005	USA	Community	63	IG 76.5(7.7), CG 77.8 (6.2);	IG: 19.3; CG: 19.4;	RCT	Galantamine 8 mg BD	8 weeks	Donepezil 10mg	Wrist actigraphy; PSQI;	SE; NNA; No. of wake bouts; Mean nocturnal wake bout duration; PSQI; PSQI Sleep medication use;	No significant effect on sleep.
Asayama [156]	2003	Japan	Hospital	20	79.2(6.4)	MMSE: IG:12.6(7.0), CG: 10.3(7.5); ADAS-Cog: IG:39.8 (35.6), CG:39.7(17.1)	RCT	Melatonin 3 mg	4 weeks	Placebo	Wrist actigraphy	NTST; Night Mean Activity Count; DTST; Day Mean Activity Count.	Significant increase in night-time sleep duration and decrease in night-time activity counts. Significant improvement in ADAS cognitive and non-cognitive assessments.
Baandrup [69]	2021	Denmark	NH	24	IG: 82.2; CG: 85.7	Inclusion criterion: MMSE ≤24	RCT	Intelligent Lighting System	4 weeks	Usual care	Wrist actigraphy	IS; IV; RA;	Significant decrease in intra-daily variability during the first half of the intervention period was not sustained.
Bademli [117]	2019	Turkey	NH	60	IG: 72.24 (7.16); CG: 70.67 (8.34)	IG: 23.27 (2.17); CG:23.42 (1.07)	RCT	Physical Activity Program	20 weeks	Usual care	PSQI	PSQI	Significant improvement in subjective sleep quality (PSQI) and cognition (MMSE).
Blytt [220]	2018	Norway	NH	106	85.5(7.3)	7.6(6.0)	RCT	Analgesia	1 week	Placebo	Wrist actigraphy	TST; SE; SoL; WASO; Early Morning Awakening; Number of Bouts Awake;	Significant improvement in sleep efficiency, sleep onset latency and early morning awakening.
Brunetti [164]	2014	Italy	Community	25	63.04(8.9)	Inclusion criterion: Diagnosis of MCI	RCT Crossover	Rivastigmine 4.6mg	30 days	Placebo	Stiasny-Kolster Scale	Frequency of RBD episodes;	Significant reduction in frequency of RBD episodes.
Burns [70]	2009	UK	NH	48	IG: 84.5; CG: 82.5;	IG: 6.9(5.3); CG: 5.1(5.6);	RCT	Bright Light Therapy 10,000 lux	Two weeks	Standard light	Wrist actigraphy	NTST; M10; L5;	No significant change in sleep.

Cai [148]	2022	China	NH	75	80(9.3)	IG: MoCA 20, MMSE 26;<br>CG: MoCA 21, MMSE 26;	RCT	Mindfulness	8 weeks	Health Education Group	PSQI: ISI; AIS;	PSQI; ISI; AIS;	Significant improvement in sleep (PSQI, ISI, AIS), cognition (MMSE, MoCA) and mood (SAS,PSS).
Camargos [182]	2014	Brazil	Geriatric Outpatients	36	81.0(7.5)	IG: 11.4; CG: 11.0;	RCT	Trazadone 50mg	14 days	Placebo	Wrist actigraphy	NTST; WASO; NNA; DTST; Number of Daytime Naps: Night % sleep;	Significant improvement in night-time total sleep time and percentage sleep.
Cassidy-Eagle [141]	2018 & 2016	USA	Independent Living Facilities	28	89.36	MoCA:<br>IG: 23.64(0.8);<br>CG: 23.5(1.01);<br>DKEFS3SS:<br>IG: 8.92(5.09),<br>CG: 10.0(4.15);	RCT	CBTi	7 weeks	Nutrition Class	ISI and wrist actigraphy	ISI; SoL; WASO; NTST; SE;	Significant improvement in sleep efficiency, total sleep time, sleep onset latency, wakefulness after sleep onset, and subjective sleep quality (ISI). Significant improvement on a measure of executive function (D-KEF Colour Word Interference Test), without further improvements in cognition.
Chan [138]	2016	China	Community Centre	52	80.6(7.12)	23.1(3.2)	RCT	Tai Chi Qigong	2 months	Health Education Group	PSQI	PSQI; PSQI domains	Significant improvement in subjective sleep quality (PSQI).
Chen [221]	2021	China	Hospital	60	IG: 71.3(6.69),<br>CG: 74.27(1.35);	IG: 13.73(3.25);<br>CG: 13.47(2.97);	RCT	MDT Assessment of Sleep Quality directing treatment according to set criteria;	24 weeks	Usual care	PSQI	PSQI	Significant within-group improvement in subjective sleep quality (PSQI) and in caregiver mood (HAMD).
Chong [125]	2006	USA	Community	39	IG: 77.7(6.9),<br>CG: 77.9(7.3);	IG: 24.2(3.1);<br>CG: 25.4(3.2)	RCT	CPAP	6 weeks	Sham CPAP	ESS; PSG;	ESS; Respiratory Disturbance Index;	Significant improvement in daytime sleepiness (ESS) and respiratory disturbance index.
Cimenser [112]	2021	USA	Community	31	IG: 66.5(8.0);<br>CG: 73.5(6.6);	IG: 19.9(2.8);<br>CG: 18.5(2.7)	RCT	40Hz Gamma Sensory Stimulation	24 weeks	Sham sensory stimulation	Wrist actigraphy	Nighttime active duration; Nighttime rest duration;	Significant reduction in night-time active duration. While the functional ability (ADCS-ADL) of the control group declined significantly, that of the intervention group did not.
Connell [118]	2007	USA	NH	20	79.7(8.3)	Total: 15.3(8.4);<br>IG: 11.7(8.51);<br>CG: 18.9(6.9);	RCT	Outdoor activity program	10 days	Indoor activity program	Wrist actigraphy	NNA; Longest duration of continuous sleep; NTST;	Total sleep time improved significantly in both groups. Longest duration of continuous sleep improved significantly in the outdoor exercise group.

Cooke [122]	2009	USA	Community	52	77.8(7.3)	Total: 25.3(2.9); IG: 24.3(2.8); CG: 24.8(4.2);	RCT	CPAP	3 weeks	Placebo CPAP	PSG	%Stage 1; %Stage 2; % Stage 3; %Stage 4; %REM; WASO; Sleep period; Sleep Onset; SE; NTIB; NTST; Arousal Index(total no of arousals per hour);	Significant decrease in time in bed, sleep period, wakefulness after sleep onset, total sleep time, arousal index, and % stage one sleep. Significant increase in % stage 3 sleep.
Cooke [123]	2009	USA	Community	10	75.7(5.9)	22.6(4.5)	RCT	CPAP	13 months	No CPAP	PSQI; ESS;	PSQI; ESS;	Large effect size demonstrating improvement in subjective sleep quality (PSQI) and less deterioration in mood (CSDD) and daytime sleepiness (ESS). Large effect size demonstrating improvements in cognition (WAIS), Wisconsin Card Sorting Test [WCST], Stroop, FAS letter fluency).
Costa [127]	2023	Canada	Outpatient Clinic	171	69.8(10.6)	MoCA: 22.4(4.22); MMSE: 25.8(3.47);	Retrospective study	CPAP	191 days (mean)	Usual care	ESS	ESS	Significant improvement in cognition (MoCA and MMSE) despite no significant improvement in daytime sleepiness.
Cremascoli [71]	2022	Italy	Outpatient Clinic	16	73 median	**Median:** IG: 19.20, CG: 26.00;	RCT	Bright Light Therapy 10,000 lux	4 weeks	Dim Light 50 lux	Wrist actigraphy; PSQI; ESS;	TST; SE; WASO; Sleep Fragmentation Index; mid-sleep time; IS; IV; falling asleep time; Dim Light Melatonin Onset; Phase-angle DLMO-bedtime; Phase-angle DLMO-falling asleep time; PSQI; ESS;	No significant change in sleep efficiency or duration, as measured by actigraphy, but there was a non-significant trend towards improvement in subjective sleep quality and a significant improvement in cognition (MMSE).
Cruz-Aguilar [154]	2013	Mexico	Hospital	8	65	Inclusion criterion: MMSE 10-22	Crossover trial	Melatonin 5mg	2 nights	Placebo	PSG	Night % sleep; N1 latency; N2 latency; Delta latency; REM latency; NTWT; N1 time; N2 time; Delta time; REM time;	Significant decrease in latency to stage 2, delta and REM sleep on the first night of melatonin administration.

Cruz-Aguilar [155]	2018 & 2021 & 2023	Mexico	Hospital	8	65.62(2.87)	Inclusion criterion: Diagnosis of mild-moderate Alzheimer's Dementia	RCT Crossover	Melatonin 5mg	1 night	Placebo	PSG	SoL; Relative Power of: Delta; Theta; Alpha 1; Alpha 2; Beta 1; Beta 2. Interhemispheric correlation during: Delta; Theta; Alpha 1; Alpha 2; Beta 1; Beta 2. Relative Power and Coherence of: Delta, Theta, Alpha1, Alpha2, Beta1, Beta2, Gamma in each of NREM1, NREM2, NREM3, REM; NREM1 Latency; NREM2 Latency; NREM3 Latency; REM Latency;	Significantly decreased latency of all stages of sleep. Several significant changes in relative EEG power and coherence.
Cumbo [165]	2014	Italy	Community	177	78.5	Memantine: 16.4(3.0); Donepezil: 16.3(2.9); Rivastigmine: 16.4(2.9); Galantamine: 16.2(3.1);	RCT	4 Groups: Memantine 20mg; Rivastigmine 12mg; Donepezil 10mg; Galantamine 24mg;	12 months	See Intervention	NPI; BEHAVE-AD;	NPI-sleep; BEHAVE-AD-diurnal rhythm;	Memantine, Rivastigmine and Donepezil produced significant improvements in both NPI and BEHAVE-AD total scores, with insignificant improvements in the sleep and diurnal rhythm sub-domains.
Cummings [222]	2015	USA	Outpatient Clinics, assisted living and NH	220	IG: 77.8(8.0), CG: 77.8(7.2);	IG: 17.4(6.0); CG: 17.2(5.8)	RCT	Dextrometorphan-Quinidine 20/10mg	10 weeks	Placebo	NPI	NPI-sleep;	Significant improvement in sleep (NPI-sleep domain), behaviour (NPI), mood (CSDD) and caregiver strain index.
deOliveira [119]	2019 & 2021	Brazil	Outpatient Clinic	54	77.4(7.5)	14.5	RCT	Tailored Activity Program	3 months	Psychoeducation	NPI	NPI-sleep;	Significant improvement in caregiver burden and several NPI domains but only insignificant improvement in sleep disturbance.
Dimpfel [223]	2020	Germany	Community	16	63.81(2.04)	Inclusion criterion: Diagnosis of MCI	Crossover trial	Adaptra Forte (Supplement)	4 weeks	Placebo	SF-B/R	Sleep Quality; Feeling refreshed after sleep; Psychic well-balanced feeling in the evening; psychic exhausted feeling in the evening; psychosomatic symptoms during sleep; dream recall; sleep-wake regulation;	Significant improvement in subjective sleep quality and in concentration (d2-Test).

Dowling [72]	2005	USA	NH	70	84(10)	7(7)	RCT	Morning Bright Light	10 weeks	Indoor light or Evening Bright Light	Wrist actigraphy	Night % sleep; NTST; NTWT; NNA; Duration of Night Awakenings; Night Mean Activity Score; DTWT; Day Mean Activity Score; R-square(cosinor); Amplitude; Acrophase;	No significant change in night-time sleep or daytime wake, but rhythmicity improved as demonstrated by a more stable acrophase.
Dowling [96]	2008	USA	NH	50	86(8)	9.3(7.9)	RCT	Morning Bright Light + Melatonin 5mg	10 weeks	Morning Bright Light + Placebo; Indoor Light;	Wrist actigraphy	NTST; Night Sleep Bout Duration; Night Wake Bout Duration; NNA; DTST; Duration of daytime naps; Day Wake Bout Duration; Number of daytime naps; Day Total Activity Counts; Day Sleep Time/Night Sleep Time; Amplitude; Acrophase; R-squared (cosinor); IS; IV; L5; M10; RA;	No significant effects on night-time sleep. Significant reduction in duration of daytime sleep, with significant increase in daytime activity and improvements in day/night sleep ratio and rest-activity rhythm.
Falck [93]	2020	Canada	Community	96	IG: 72(6), CG: 74(5);	IG: 28.0(1.5); CG: 28.3(1.3);	RCT	Multimodal lifestyle intervention: Chronotherapy (Sleep Hygiene Classes, BLT, Counselling)	24 weeks	Wait-list	Wrist actigraphy; PSQI; Consensus Sleep Diary;	SE; NTST; SoL; Sleep Fragmentation Index; WASO; PSQI; IS; IV; L5; M10; RA;	Significant improvement in subjective sleep quality (PSQI). No significant improvement in objectively measured sleep parameters.
Fei [224]	2023	China	Hospital	42	IG: 76.4, CG: 75.3;	MMSE: IG: 21.75(2.57), CG: 20.85(2.25); MoCA: IG: 19.80(1.85), CG: 19.80(1.15);	RCT	Probiotic	12 weeks	Placebo	PSQI	PSQI; PSQI domains	Significant improvement in subjective sleep quality (PSQI) and cognition (MMSE, MoCA).
Femia [225]	2007	USA	Community	201	IG: 60.2; CG: 66.4;	IG: 14.6; CG: 14.2	Quasi-experimental	Adult Day Services	2 months	Usual care	Daily Record of Behaviour - Sleep Disturbances category	Daily Record of Behaviour - Sleep Disturbances category	Significant improvement in sleep.
Fernandez [226]	2023	USA	Community	82	IG: 72.13(8.13), CG: 74.13(5.86)	MoCA: IG: 17.42(3.77); CG: 19.25(3.31);	RCT	SYN120 (100mg QDS)	16 weeks	Placebo	Scales for Outcomes in Parkinson's Disease - Sleep Scale (SCOPA-Sleep)	SCOPA-Daytime Sleepiness; SCOPA-Nighttime Sleep;	No significant effect on sleep. Significant improvement in NPI apathy/indifference sub-domain.

Figueiro [80]	2019	USA	Assisted Living	46	85.1(7.1)	14.7(4.3)	RCT Crossover	Tailored Lighting Intervention	4 weeks	Controlled Tailored Lighting	Wrist actigraphy; PSQI;	IS; IV; PSQI; TST; Night % sleep; SoL; Number of daytime naps;	Significant improvement in subjective sleep quality (PSQI) and in intra-daily variability measured by actigraphy. Significant improvements in mood (CSDD) and agitation (CMAI).
Fleischhacker [179]	1986	Austria	Hospital	20	IG: 75.3(8.4); CG: 79.6(7.6);	Inclusion criterion: Diagnosis of Senile Dementia of Alzheimer's Type	RCT	Memantine 20 - 30mg	5 weeks	Placebo	Geriatric Rating Scale - sleep domain	Geriatric Rating Scale - sleep domain	No significant improvement in sleep.
Fontana Gasio [73]	2003	Switzerland	NH	13	IG: 86.8(4.5), CG: 83.0(5.2);	13.9	RCT	Dawn-Dusk Simulation Light	3 weeks	Dim red light	Wrist actigraphy; Nursing Sleep Logs;	IS; IV; RA; L5; Onset of L5; M10; Onset of M10; NTIB; Sleep Start Time; Sleep End Time; NTST; SE; NNA; SoL; Number of Immobile Phases; Number of one minute immobility epochs; Mean Activity Counts;	No significant change apart from phase advance of time of onset of the least active five hour period.
Foster [227]	1989	USA	Community	10	67(5)	22	RCT Crossover	RS-86 (cholinergic agonist)	4 weeks	Placebo	PSG;	REM Latency; Total REM duration; %REM; NTST; Number of REM periods; Time between first and second REM periods;	Significant decrease in REM latency, with significant increase in duration and percentage of REM sleep.
Friedman [77]	2011	USA	Community	59	77.9(8.1)	22.1(4.7)	RCT	Bright Light Therapy	2 weeks	Dim light	Wrist actigraphy; Daily caregiver sleep report; ESS; Blake-Gomez Sleep Hygiene Questionnaire ; Global Sleep Assessment Questionnaire ;	WASO; TIB; NTST; SE; ESS; Global Sleep Assessment Questionnaire;	Significant decrease in total sleep time and time in bed, which was associated with a significant decrease in sleep efficiency and a significant increase in wakefulness after sleep onset.
Froggatt [228]	2020	England	NH	32	69.9(13.7)	Inclusion criterion: Diagnosis of advanced dementia	Cluster RCT	Namaste Care	4 weeks	Usual care	Wrist actigraphy	Sleep/Wake Ratio; NTST; SE; WASO; IS; IV;	No significant effect on sleep reported.
Gattinger [229]	2017	Switzerland	NH	52	87.5(7.1)	Inclusion criterion: cognitive impairment	Cluster RCT	Mobility Monitoring System	22 weeks	Usual care	PSQI; Essen Questionnaire on Age and Sleepiness; Mobility Monitor;	PSQI; Essen Questionnaire on Age and Sleepiness; TIB; Max Duration of immobility; Number of bed exits;	Significant improvement in subjective sleep quality (PSQI).

Gehrman [126]	2009	USA	NH	41	82.9(7.0)	5.8(5.6)	RCT	Melatonin 10mg	10 days	Placebo	Wrist actigraphy	NTST; SE; NTWT; Night % awake; DTST; Day % Sleep; Number of daytime naps; Duration of daytime naps; Amplitude; Acrophase;	No significant improvement in sleep parameters.
Grove [230]	2014	International	Community	196	IG: 71.6(8.79), CG: 72.0(8.59);	IG: 20.0(2.66); CG: 19.9(2.54);	RCT	GSK239512	16 weeks	Placebo	PSQI; ESS;	PSQI; ESS;	No significant effect on sleep. Significant improvement in episodic memory.
Harris [135]	2012	USA	NH	40	IG: 84.6(6.11), CG: 87.5(6.78);	10.58(7.31)	RCT	Slow Stroke Back Massage	2 nights	Usual care	Wrist actigraphy	NTST; SoL; SE; WASO; DTST; Day/Night Sleep Ratio;	No significant improvement in sleep parameters.
Hastings [231]	2021	USA	Community	40	72.4(6.1)	Modified Telephone Interview for Cognitive Status: 27.6(2.6)	RCT	Care Management Intervention via video	12 weeks	Care Management Intervention via Telephone	PSQI	PSQI	No significant effect on sleep.
Herring [185]	2020	International	Outpatient Clinic	285	IG: 69.6(8.7), CG: 69.1(8.5);	IG: 22.5(3.0); CG: 22.3(3.3);	RCT	Suvorexant 20mg	4 weeks	Placebo	PSG; Wrist actigraphy; Caregiver rating of sleep quality; SDI; Clinician's Global Impression of Insomnia;	NTST; WASO; SE; SoL; NNA; Number of Arousals; %N1; %N2; %N3; %REM; REM latency; NTIB; Caregiver report of sleep quality; subjective sleep quality; subjective report of waking earlier than planned;	Significant improvement in sleep efficiency, total sleep time and wakefulness after sleep onset. Significant improvement in sleep, as rated by both care partners and clinicians.
Hickman [74]	2007	USA	Psychiatric Hospital & NH	66	<65 years: 9.1%; 65-79 years: 42.42%; ≥80 years: 48.48%	Inclusion criterion: Diagnosis of dementia	Cluster unit crossover trial	3 Groups: Morning Bright Light; Evening Bright Light; All Day Bright Light;	3 weeks	Standard Light;	Cornell Scale for Depression in Dementia	Cornell Scale for Depression in Dementia-Sleep Disturbance	No significant effect on sleep.
Hjetland [83]	2021	Norway	NH	69	84	6.4(6.7)	Cluster RCT	Bright Light Therapy	24	Standard Light;	SDI; Wrist actigraphy;	TST; NTST; DTST; WASO; Night % sleep; SDI;	Significant improvement in subjective sleep quality (SDI) but no significant effect on sleep when measured by actigraphy.

Hopkins [78]	2017	UK	NH	80	85.8(7.5)	19(6)	RCT Crossover	High colour temperature blue-enriched white light	4 weeks	Low colour temperature white light	Karolinska Sleepiness Scale; PSQI; Wrist actigraphy;	Cosinor Amplitude; Mesor; Acrophase; IS; IV; L5; L5 onset; M10; M10 onset; Amplitude; RA; NTST; Night % Sleep; WASO; Night % awake; SoL; SE; Mean Activity Score; Mean Activity Score in Wake Periods; Mean Wake Bout Time; Fragmentation Index; Mean Wake Score; PSQI; Karolinska Sleepiness Scale;	While the peak time (acrophase) of the rest-activity rhythm was significantly advanced, there was significant decrease in total sleep time and sleep efficiency, with significant increases in wakefulness after sleep onset. There was also a significant deterioration in subjective sleep quality (PSQI). There was a significant improvement in daytime anxiety (HADS).
Hozumi [105]	1996	Japan	Hospital	27	74.2	Inclusion criterion: Diagnosis of dementia	RCT	Transcranial Electrostimulation	2 weeks	Placebo TCES	Nursing Sleep Diary; Doctor clinical evaluation of sleep disorder;	Nursing Sleep Diary; Doctor clinical evaluation of sleep disorder;	Sleep improved in both groups. No significant between-group improvement in sleep.
Hsiao-Mei [145]	2016	Taiwan	NH	44	IG: 83.9, CG: 75.6;	IG: 13.87(3.78); CG: 14.19(5.28);	RCT Crossover	Individualised Learning Therapy	12 weeks	Usual care	NPI	NPI-sleep	Significant improvement in sleep (NPI-sleep domain), cognition (MMSE), and behaviour (NPI).
Hu [109]	2022	China	Hospital	88	TMS-DCS:: 79.33(6.24); Single rTMS: 76.86(6.07); Single tDCS: 77.1(6.88); CG: 75.33(5.73);	TMS-DCS: 14.52(3.04); Single rTMS: 13.52(2.94); Single tDCS: 13.29(3.45); CG: 15.05(3.12);	RCT	TMS-DCS; TMS; DCS;	4 weeks	Sham stimulation	NPI; PSQI;	PSQI; NPI-sleep;	Significant improvements in subjective sleep quality (PSQI), cognition (MMSE), and neuropsychiatric symptoms (NPI), including in the sleep domain specifically.
Huang [124]	2021	China	Outpatient Clinic	97	73	Inclusion criterion: Diagnosis of Alzheimer's Dementia. All participants had MoCA 12-26.	Prospective longitudinal	Continuous Positive Pressure Ventilation + Donepezil	6 months	Donepezil	PSQI;	PSQI;	Significant improvements in subjective sleep quality (PSQI), cognition (MoCA), mood (HAMD), anxiety (HAMA), and blood-based biomarkers of neurodegeneration (AB42/40 ratio, Tau-pT181).

Huo [187]	2022	China	Hospital	96	IG: 67.38(17.98), CG: 67.31(17.89);	Inclusion criterion: Diagnosis of Alzheimer's Dementia.	RCT	Eszopiclone 3mg	4 weeks	Alprazolam 400mcg	PSQI; EEG; BEHAVE-AD;	PSQI domains; BEHAVE-AD - diurnal rhythm; SoL; REM Latency; NTST; SE; NREM time; REM time; Sleep cycle;	Significant improvements in objective and subjective sleep measures, including in sleep latency, sleep efficiency, total sleep time, time in NREM, time in REM, and in all domains of the PSQI. Significant improvements in cognition (MMSE), behaviour (BEHAVE-AD), and function (ADLs),
Ito [232]	2001	Japan	Community	28	78.3	Inclusion criterion: Diagnosis of Alzheimer's-type Dementia	RCT	Bright Light Therapy + Vitamin B12	8 weeks	Bright Light Therapy	Wrist actigraphy	Night % sleep; Day % sleep; Number of daytime naps; NNA;	Significant decrease in daytime naps and sleep percentage. No significant effect on night-time sleep.
Jean-Louis [157]	1998	USA	Community	10	68.8(15.8)	MMSE median 28	RCT Crossover	Melatonin 6mg	10 days	Placebo	Wrist actigraphy; Stanford Sleepiness Scale; Sleep Log;	NTST; SE; SoL; WASO; NTWT; Transition from sleep to wakefulness; Amplitude; Cosinor fit;	Significant increase in amplitude, with significant decreases in sleep onset latency and transitions from sleep to wakefulness. Significant improvements in mood and delayed recall (ADAS-Cog).
Joranson [131]	2021	Norway	NH	60	IG: 83.9(7.17), CG: 85.2(6.73);	Inclusion criteria: Diagnosis of dementia/MMSE<25.	Cluster RCT	Paro Robotic Seal	12 weeks	Usual care	Wrist actigraphy;	SE: NTST; WASO; Night awakenings longer than 5 minutes;	Significant improvement in total sleep time, sleep efficiency, wakefulness after sleep onset and number of night-time awakenings longer than five minutes.
Keramtinejad [146]	2019	Iran	Community	108	IG: 63.42(4.42), CG: 64.47(5.65);	CDR: IG: 4.14(1.85); CG: 3.55(1.9);	RCT	Cognitive Training	8 weeks	Wait list	PSQI;	PSQI; PSQI domains	Significant improvement in subjective sleep quality (PSQI) and cognition (CDR).
Kim [233]	2016	South Korea	NH	30	78.5	IG: 10.5(9.4); Placebo: 12.3(8.4); CG: 9.6(8.7);	RCT	Footbath (40C)	4 weeks	Placebo: Footbath (36.5C); Control: Usual care;	Wrist actigraphy; SDI;	TST; SE: SoL; SDI;	Significant improvement in total sleep time and sleep efficiency.
Kim [84]	2021	South Korea	Outpatient Clinic	25	IG: 77.36(5.79); CG: 78.55(7.71);	IG: 16.36(5.09; CG: 16.9(4.91)	RCT	Bright Light Therapy 9 hrs post DLMO	2 weeks	Blue-attenuated bright light therapy	Wrist actigraphy; PSQI;	NTST; NTIB; Sleep Onset time; Mid Sleep time; SoL; WASO; SE; Fragmentation Index; PSQI;	Significant improvement in subjective sleep quality (PSQI) and caregiver burden (Zarit). No significant improvement in objectively measured sleep parameters.
Kim [136]	2021	South Korea	Community	60	IG: 69.4(7.3); CG: 72.5(8.4);	IG: 22.9(3.5); CG: 23.9(3.8);	RCT	Electrical Automatic Massage chair	12 months	Usual care	PSQI;	PSQI, plus the following PSQI domains: sleep quality, SoL, NTST, sleep disturbance.	Significant improvement in subjective sleep quality (PSQI - sleep quality question).

Kolberg [75]	2021	Norway	NH	69	Median 84	Median: 4	Cluster RCT	Bright Light Therapy	24 weeks	Usual care	Wrist actigraphy	IS; IV; L5; M10; RA; Amplitude; MESOR; Acrophase; Nadir; Alpha; Beta; Pseudo-F statistic;	Significant advance of acrophase after 16 weeks, but no other significant effect on rhythmicity.
Korucu [167]	2018	Turkey	Hospital	35	IG: 753.5(6.66), CG: 73.93(7.62);	19.25(3.11)	Prospective observational	Donepezil	1 month	Rivastigmine	PSQI	PSQI;	No significant effect on sleep.
Kouzuki [234]	2020	Japan	Outpatient Clinic	43	Median: IG: 82, CG: 78 in 0.5%, 76 in 0.1%;	Inclusion criteria: Diagnosis of Alzheimer's Dementia/Diagnosis of MCI.	RCT	Aroma oil/bath salt 1%;	24 weeks	Aroma oil/bath salt 0.5% and 0.1%;	PSQI;	PSQI; PSQI domains;	No significant effect on sleep.
Ladenbauer [104]	2017	Germany	Outpatient Clinic	22	71.2(8.79)	28.3(1.4)	RCT Crossover	Transcranial Electrostimulation	90 minute nap;	Sham electrostimulation;	PSG; Tiredness Symptoms Scale;	Frontal and Centroparietal slow oscillation power; functional coupling of slow oscillations and spindles (phase-amplitude coupling); Frontal and centroparietal fast spindle power; Frontal slow spindle power; TST; WASO; %N1; %N2; %N3; %N4; %REM;	Significant enhancement of frontal and centroparietal cortical slow oscillation power and fast spindle power. Significant enhancement of frontal slow spindle power. Improved slow oscillation and spindle synchronisation. Significant improvement in visual memory, but no significant improvement in verbal memory, procedural memory or location memory.
Larsson [178]	2010	International (Norway, Sweden, UK)	Outpatient Clinic	57	IG: 76.4(6.5); CG: 76.3(5.0);	IG: 20.2(3.7); CG: 19.6(4.4);	RCT	Memantine 10mg BD	24 weeks	Placebo	Stavanger Sleep Questionnaire; ESS;	Stavanger Sleep Questionnaire question on probable RBD: physically active during night sleep?; ESS;	Significant decrease in physical activity during sleep.
Leonpacher [97]	2016	USA & Canada	Community	186	Not reported	Inclusion criterion: Diagnosis of probable Alzheimer's disease	RCT	Citalopram 30mg + psychosocial intervention	9 weeks	Placebo	NPI	NPI-sleep	No significant effect on sleep (NPI-sleep domain). Significant improvement in anxiety, delusions and irritability (NPI domains).
Li [235]	2017	USA	NH	28	86.45(6.9)	Inclusion criterion: Diagnosis of dementia	Cluster RCT	Person-centred dementia care	3 months	Usual care	Wrist actigraphy	DTST; Day % sleep; NTST; Night % sleep; Arousal Index;	Significant increase in night-time sleep and decrease in daytime sleep.
Linander [76]	2020	Denmark	NH	40	81.4(8.26)	All participants were stated to have dementia	Crossover trial	CaLED (circadian lighting)	8 weeks	Conventional Lighting;	PSQI; ESS;	PSQI; ESS;	No significant effect on sleep.

Liu [87]	2022	Taiwan	NH + community	35	IG: 83.9(7.1), CG: 80.2(7.2);	Inclusion criterion: Diagnosis of dementia	Prospective longitudinal	Bright Light Therapy	8 weeks	Dim light	Wrist actigraphy	SE; NTST; NTWT; NNA; Time of sleep onset; Time of sleep offset;	Significant improvement in sleep efficiency and total sleep time, with significant advance in sleep onset time and delay in sleep offset time.
Livingstone [98]	2019	UK	Community	62	IG: 80.4(9.0), CG: 79.6(7.0);	Inclusion criterion: Diagnosis of dementia	RCT	DREAMS-START	3 months	Usual care	Wrist actigraphy; ESS; SDI;	ESS; SDI; SE; NTST; NTWT; Average bedtime; Average time of falling asleep; Average time of waking up; Average time of getting up; NTIB; IS; IV; RA; L5; L5 start; M10; M10 start; SE of core night hours; Average sleep time of core night hours; Average wake time of core night hours;	No significant effect on night-time sleep. Significant improvement in daytime sleepiness (ESS), quality of life (DEMQoL-Proxy) and caregiver burden (Zarit).
Louzada [188]	2022	Brazil	Community	62	80.5	Zolpidem: 7.9(6.6); Zopiclone: 10.0(6.0); CG: 11.8(7.2);	RCT	Zolpidem 10mg; Zopiclone 7.5mg;	14 days	Placebo	Wrist actigraphy; NPI;	Main nocturnal sleep duration; WASO; NNA; Daytime TST; Number of daytime naps; NPI-sleep;	Zopiclone produced significant improvements in main night-time sleep duration, wakefulness after sleep onset and number of night-time awakenings, with significant improvement in behaviour as well (NPI). Zolpidem produced significant improvements in wakefulness after sleep onset and number of night-time awakenings. While neither zopiclone nor zolpidem had a significant effect on total MMSE score, both resulted in significant deteriorations in other aspect of cognition.
Lyketsos [81]	1999	USA	NH	15	80.8(8.7)	6.4(6.8)	RCT Crossover	Bright Light Therapy	4 weeks	Dim light	Sleep Log; BEHAVE-AD;	NTST; BEHAVE-AD-diurnal rhythm;	Significant improvement in mean nocturnal hours of sleep.
Markowitz [175]	2003	Internationa l	Community	386	75(7.5)	19.8(3.6)	RCT	Galantamine 12mg BD	3 months	Placebo	PSQI; NPI;	PSQI; PSQI domains; PSQI uncounted questions; NPI-sleep;	No significant effect on sleep.

McCurry [236]	2003	USA	Community	22	77.4(6.6)	10.7(7.8)	RCT	Caregiver Sleep Hygiene Programme	2 months	General caregiver education	Daily sleep logs	Consistent bedtime; Consistent waking time; Nap restriction;	Significant improvements in maintaining consistent bed times and rising times, and in restricting naps and walking.
McCurry [94]	2005	USA	Community	36	IG: 62.8(15.3), CG: 63.7(16.7);	11.8(8.4)	RCT	NITE-AD (Walking, Light and sleep education)	2 months	General caregiver education	Wrist actigraphy; ESS;	Bedtime; Rising Time; NTIB; NNA; NTST; Night % sleep; DTST; ESS; Wake Index (Wakes per hour); WASO; NTWT; Acrophase; Amplitude; MESOR;	Significantly reduced night-time total wake time, with significantly lower levels of depression (Revised Memory & Behaviour Problems Checklist).
McCurry [67]	2011	USA	Community	132	Walking: 82.2(8.5); Light: 80.6(7.3); NITE-AD: 80.0(8.2); CG: 81.2(8.0);	Walking: 19.2(7.7); Light: 17.9(7.0); NITE-AD: 19.1(5.8); CG: 18.7(6.9);	RCT	1. Walking, 2. Light, 3. Walking, Light and Sleep Education (NITE-AD).	2 months	Non-directive dementia care support	Wrist actigraphy; SDI;	NTWT; Night % Sleep; NNA; NTST; NTIB; DTST; SDI;	All three intervention groups showed significant improvements in night-time total wake time. The NITE-AD group had significant improvement in objectively measured night-time sleep percentage.
McCurry [237]	2012	USA	Community	47	86.6(7.2)	8.1(7.6)	RCT	Sleep Education Programme	4 weeks	Usual care	Wrist actigraphy; ESS;	Bedtime; Rising Time; NNA; NTIB; NTWT; NTST; Night % sleep: DTST; ESS;	Significant improvement in mood (CSDD) post-treatment. Significant improvement in night-time total sleep time and percentage sleep at six month follow-up.
McPhillips [238]	2022	USA	Community	20	69.1(7.45)	MoCA: 21.1(2.49)	RCT	Digital Assisted Relaxation Therapy	2 weeks	Digital Sleep Education Program	PSQI; ISI; ESS;	PSQI; ISI; ESS;	Significant within-group improvement in subjective sleep quality (PSQI and ISI).
Meguro [189]	2004	Japan	NH	34	IG: 78.5; CG: 77.1;	IG: 12.5; CG: 11.8;	RCT	Risperidone 1mg	4 weeks	Usual care	Sleep log;	NTST; DTST;	Significant increase in night-time sleep and decrease in daytime sleep.
Mishima [82]	1994	Japan	Hospital	14	75	All participants were stated to have dementia	Prospective observationa l	Bright Light Therapy	4 weeks	Usual care	Nursing sleep and behaviour diary;	NTST; DTST; Serum melatonin;	Significant increase in total sleep time and nocturnal sleep time, with significant decrease in daytime total sleep time.
Mishima [89]	1998	Japan	Hospital	22	Vascular dementia: 81; Alzheimer's Dementia: 78	Vascular dementia: 8; Alzheimer's Dementia: 9;	RCT Crossover	Bright Light Therapy	2 weeks	Dim Light	Wrist actigraphy	Mean Nighttime Activity; % Nighttime Activity;	Significant reduction in night-time activity levels, but only in those with vascular dementia, not in those with Alzheimer's.
Mok [168]	2007	China	Outpatient Clinic	40	IG: 75.7(5.1), CG: 74.1(6.6);	IG: 13.0(4.2); CG: 13.4(5.9);	RCT	Rivastigmine 6mg	26 weeks	Placebo	NPI	NPI-sleep	No significant effect on sleep.

Moline [186]	2021	USA, Japan, UK	Community	62	CG: 75.3(6.2); IG 2.5mg: 76.5(6.3); IG 5mg: 76.9(8.0); IG 10mg: 71.8(7.1); IG 15mg: 71.9(6.1);	Inclusion criteria: Diagnosis of Alzheimer's Dementia/MMSE10-26.	RCT	4 Groups: Lemborexant 2.5mg, Lemborexant 5mg, Lemborexant 10mg, Lemborexant 15mg.	4 weeks	Placebo	Wrist actigraphy; SDI;	L5; L5 start time; M10; RA; IS; IV; Wake Efficiency; Wake Fragmentation Index; Number of daytime naps; Duration of daytime naps; DTST; Night % sleep; Sleep Fragmentation Index; NTST; Mean number of nocturnal wake bouts; Mean duration of nocturnal wake bouts; SDI (reported separately);	Significant reduction in mean activity count during least active five hours in 2.5mg, 5mg and 15mg groups. Significant improvements in relative amplitude in the 5mg and 15mg groups. Significant reduction in number of night-time awakenings in the 2.5mg and 5mg groups.
Moraes [173]	2006	Brazil	Outpatient Clinic	35	IG: 77.4(6.6); CG: 74.5(9.8);	ADAS-Cog: IG: 35.6(13.7); CG: 39.0(18.5);	RCT	Donepezil 10mg	6 months	Placebo	PSG	NTST; Night % sleep; SoL; REM latency; REM density; %REM; %Stage1; %Stage2; %SWS; AHI; Arousals per hour; periodic leg movements per hour; mean oxygen saturation; Delta, theta, alpha, beta: Overall, Frontal, Temporal, Centroparietal, Occipital. Slowing Ratio: overall, frontal, temporal, centroparietal, occipital;	Significant increase in REM percentage, with significant improvement in cognition (ADAS-Cog). Significant decrease in overall theta frequency band, and in frontal delta and theta bands, with significant decrease in occipital slowing ratio.

Moraes [174]	2008	Brazil	Outpatient Clinic	23	IG 76.8(6.2), CG 72.6(11.0)	ADAS-Cog: IG: 34.5(15.8); CG: 29.3(17.3); MMSE: IG: 19(3.6); CG: 17.2(7.8);	RCT	Donepezil 10mg	3 months	Placebo	PSG;	NTST; Night % sleep; SoL; REM latency; WASO; %REM; %Stage 1; %Stage 2; %SWS; NTIB; Microarousal Index; Non-respiratory microarousal index; Respiratory microarousal index; Apnoea Hypopnoea Index; Obstructive AHI; Central AHI; Mixed AHI; REM AHI; NREM AHI; Average oxygen saturation; %sleep time with oxygen sats <90%; Number of desaturation events; Lowest oxygen saturation;	Significant increase in REM percentage, with significant improvements in Apnoea-Hypopnoea Index, oxygen saturation and cognition (ADAS-Cog).
Morales-Delgado [158]	2018	Mexico	Outpatient Clinic	40	IG: 82.2(5.8), CG: 83.1(7.4)	Median: 18	RCT	Melatonin 5mg	8 weeks	Placebo	PSQI; NPI;	PSQI; NPI-sleep;	Significant improvement in the sleep sub-domain of NPI, with significant within-group improvement in subjective sleep quality (PSQI).
Moretti [166]	2008	Italy	Outpatient Clinic	200	73.34	Multi-infarct dementia: 18.64(2.13); subcortical vascular dementia: 20.72(1.97);	RCT	Rivastigmine 6mg	14 months	Nimodipine 60mg	BEHAVE-AD;	BEHAVE-AD-diurnal rhythm;	Significant improvement in sleep (BEHAVE-AD sub-domain), behaviour (BEHAVE-AD and Ryden Aggression Scale), mood (GDS).
Moyle [132]	2018	Australia	NH	415	IG: 84(8.8); Plush toy: 86(7.6); Usual care: 85(6.9);	Inclusion criterion: Diagnosis of dementia	Cluster RCT	PARO Robotic Seal	10 weeks	2 Groups: Plush toy; Usual care;	Wrist actigraphy;	Nighttime physical activity; Nighttime step count; NTST; NTIB; NTWT; DTST; DTWT; DTIB;	Significant reductions in night-time step count and daytime physical activity. No significant improvement in sleep parameters.
Münch [79]	2017	Switzerland	NH	104	78.4(9.0)	8.8(10.2)	RCT	Dynamic Lighting	8 weeks	Conventional Lighting;	Wrist actigraphy	IS; IV; RA; Salivary melatonin; L5; L5 onset; M10; M10 onset; Bedtime; Waketime; NTIB; Sleep start; Sleep end; NTST; NTWT; SE; SoL; Sleep Fragmentation Index;	Significantly later bedtime, sleep onset time and decreased time in bed. Significant improvement in mood (Observed Emotion Rating Scale) and quality of life (QUALID).

Nacu [239]	2016	Moldova	Community	410	IG: 65.1(8.8), CG: 64.9(9.4);	Inclusion criterion: Diagnosis of mild-moderate dementia	RCT	G biloba extract EGb761	24 weeks	Placebo	NPI	NPI-sleep; NPI Caregiver Distress-sleep;	Significant improvement in sleep (NPI-sleep domain), behaviour (NPI), and caregiver distress (NPI).
Naharci [169]	2015	Turkey	Outpatient Clinic	55	CG: 77.4(6.3); Rivastigmine: 81.5(6.9); Donepezil: 80.9(7.4); Galantamine: 79.1(7.7);	Cholinesterase inhibitor: 20.94(4.08); CG: 27.91(1.83);	Prospective observational	3 Groups: Galantamine/Donepezil/Rivastigmine	Mean 6.1 months	Usual care	PSQI;	PSQI	Galantamine produced a significant improvement in subjective sleep quality (PSQI).
Naismith [142]	2019	Australia	Outpatient Clinic	35	IG: 69.4(9.5), CG: 70.0(8.8);	IG: 28.1(2.7); CG: 28.5(2.1);	RCT	Sleep Well Think Well (CBT)	8 weeks	Sleep Education Material	PSQI; Wrist actigraphy; ESS;	PSQI; PSQI domains; NTST; WASO; Night % sleep; ESS;	Significant improvement in subjective sleep quality (PSQI). No significant improvement in objectively measured sleep parameters.
Nakamura [180]	2014	Japan	Outpatient Clinic	640	74.2(8.8)	9.99(2.95);	RCT (pooled analysis)	Memantine 20mg	24 weeks	Placebo	BEHAVE-AD	BEHAVE-AD-diurnal rhythm;	Significant improvement in cognition and behaviour (BEHAVE-AD). No significant improvement in sleep.
Nascimento [120]	2014	Brazil	Community	30	IG: 76.8(6.8), CG: 77.9(5.9);	IG: 14.3(5.9); CG: 13.7(7.7)	RCT	Multimodal exercise programme	6 months	Usual care	Mini-Sleep Questionnaire;	Mini-Sleep Questionnaire;	Significant improvement in sleep disturbance and performance of instrumental activities of daily living (FAQ).
Nizamutdinov [110]	2021	USA	Community	60	74.2(7.7)	IG: 22.8(2.6); CG: 23.2(1.6);	RCT	Transcranial Infra-red light helmet device	8 weeks	Sham helmet	Caregiver sleep log;	NTST	Significant improvements in cognition.
Ohman [121]	2017	Finland	Community	210	77.8	Community Exercise: 18.9(6.5); Home Exercise: 18.6(6.2); CG: 17.8(6.0);	RCT	Home Exercise Group; Community Exercise Group;	12 months	Usual community care	NPI	NPI-sleep	No significant effect on sleep.

Ouslander [99]	2006	USA	NH	230	IG: 83.5(8.71); CG: 82.9(9.3);	IG: 10.9(8.9); CG: 12.5(8.8);	Cluster RCT	Multimodal: exercise, bed restriction, consistent bedtime, noise abatement, modified continence care;	17 days	Usual care	Wrist actigraphy; Nursing observation logs; PSG (subset)	% of observations in bed during daytime; Daytime % of observations asleep; Day % sleep; Daytime Max sleep bout duration; Duration of daytime naps; Night % sleep; Night max sleep bout duration; Night mean sleep bout duration; NNA; Number of noise-related awakenings; NTIB; NTST; Time in NREM; Time in REM; SoL; WASO; SE:	Significant reduction in daytime sleep percentage, but no significant improvement in night-time sleep.
Papalambros [111]	2019	USA	Community	9	72	Inclusion criterion: Diagnosis of amnestic MCI	RCT Crossover	Acoustic Stimulation	1 night	Sham stimulation	PSG	NTST; SE; SoL; WASO; Time in Stage1; Time in Stage 2; Time in Stage 3; Time in REM; Time in Stage 2 + 3; % Stage 1; %Stage 2; % Stage 3; % REM; % Stage 2 + 3; Number of arousals; Number of arousals in Stage 2; Number of Arousals in Stage 3; Arousal Index; Arousal Index in Stage 2; Arousal index in Stage 3; Spindle amplitude; Spindle duration; Spindle frequency (Hz); Spindle density (number/min); Mean slow oscillation power; mean slow wave activity power; Mean Sigma power; Mean Theta power; Mean Beta power;	Significant enhancement of cortical slow oscillations and slow wave activity, with insignificant trend towards improvement in cognition.

Petit [240]	1993	Canada	Community	8	61.9	21.4	RCT Crossover	Tetrahydroaminoacridine (THA) 100mg	2 weeks	Placebo	PSG	SoL; SE: Night % awake; NNA; % Stage1; % Stage 2; % Stage 3 + 4; % REM; REM latency; Number of REM periods; REM efficiency; Ratio of power in slow frequencies (delta+theta) to fast frequencies (alpha+beta) was used as a measure of cortical slowing during both wakefulness and sleep in: frontal, temporal, and parietal lobes;	No significant effect on sleep.
Petrovsky [143]	2023	USA	Community	33	71.7(7.1)	Inclusion criterion: Diagnosis of dementia or self-reported cognitive impairment and CDR >0.5	RCT	Tailored Music Intervention	4 weeks	Wait-list	Wrist actigraphy; PROMIS-SF; SDI;	SoL; WASO; TST; PROMIS-SF; SDI;	Improvement in total sleep time.
Pu [133]	2021	Australia	NH	43	IG: 86.48(8.81), CG: 85.5(6.02);	IG: 7.71(7.84); CG: 11.55(8.06);	RCT	Robotic Seal	6 weeks	Usual care	Wrist actigraphy;	DTIB; DTWT; DTST; Daytime light/deep/very deep sleep time; Night step counter; NTIB; Night distance travelled; Night physical activity; NTWT; NTST; Night light/deep/very deep sleep time;	Significant increase in daytime total wake time and decrease in daytime sleep. No significant effect on night-time sleep parameters.
Reverte-Villarroya [241]	2020	Spain	Community	36	78.96(6.64)	IG: 16.92(7.48); CG: 16.53(5.02);	RCT	Caregiver Education	3 months	Usual care	NPI	NPI-sleep	Significant deterioration in cognition (MMSE), mood (GDS), and caregiver burden (Zarit). Significant improvement in sleep among those who had pre-existing behavioural & sychological symptoms of dementia.
Richards [242]	2005	USA	NH	139	79(8.4)	8.7(7.1)	RCT	Individualised Social Activity Intervention	21 days	Usual care	Wrist actigraphy	DTST; SoL; NTST; NTWT; SE; Day/Night Sleep Ratio;	Significant decrease in daytime sleep and daytime/night-time sleep ratio.

Richards [128]	2019	USA	Community	68	IG: 67.4(7.2); CG: 73.2(8.6);	IG: 28.3(1.4); CG: 28.0(2.1);	Quasi-experimental	CPAP adherence (>4 hrs/night)	1 year	CPAP non-adherence (<4 hrs/night)	ESS;	ESS	Significant increase in psychomotor/cognitive processing (digital symbol subtest of WAIS). Significant decrease in daytime sleepiness (ESS). The control group experienced a significant decrease in cognition (MMSE).
Riemersma [88]	2008	Netherlands	NH	189	85.8(5.5)	87% of participants stated to have a diagnosis of dementia	RCT, with clustering of light intervention;	Bright Light Therapy + melatonin 2.5mg; Bright Light Therapy; Melatonin 2.5mg;	Mean 15 months	Usual care	Wrist actigraphy	NTST; NTWT; SoL; SE; Nocturnal Restlessness; Mean duration of nocturnal awakenings; Mean duration of sleep bouts;	Light showed significant improvements in total sleep time, cognition (MMSE), mood (CSDD) and activities of daily living. Melatonin produced significant improvements in sleep onset latency, total sleep time and duration of uninterrupted sleep epochs, but had significant negative effect on mood (Philadelphia Geriatric Centre Affect Rating Scale). Combination light and melatonin significantly improved sleep efficiency, nocturnal restlessness, duration of nocturnal awakenings, and agitation (CMAI).
Rodriguez-Mansilla [137]	2013	Spain	NH	120	CG: 81.9(5.9); Massage: 85.8(4.9); Ear Acupuncture: 85.4(5.9);	Inclusion criterion: Diagnosis of dementia	RCT	2 Groups:<br>Ear Acupunture; Massage;	3 months	Usual care	Bespoke question	Sleep Disturbance? (Yes/No)	Both interventions produced significant improvement in sleep and behavioural disturbances.
Rosenberg [243]	2016	USA	Community	1408	73.0(8.1)	20.9(3.5)	RCT	Semagacestat 100mg/140mg	76 weeks	Placebo	NPI	NPI-sleep	No improvement in sleep.
Rozankovic [170]	2021	Croatia	Outpatient Clinic	91	IG: 71.5, CG: 72.6	Donepezil: 17.2; Memantine: 17.1;	RCT	Donepezil 10mg	2 months	Memantine 20mg	NPI	NPI-sleep	Both donepezil and memantine significantly improved scores in the NPI overall and in the sub-domain of sleep.

Savaskan [190]	2006	Switzerland	Hospital	30	Haloperidol: 82.3(2.5); Quetiapine: 81.9(1.8);	Quetiapine: 19.9(1.3); Haloperidol: 18.1(1.3)	RCT	Haloperidol	5 weeks	Quetiapine	Wrist actigraphy; NPI	NPI-sleep; TWT/wake bout (mean nocturnal wake bout duration); Mean length immobile (Mean sleep bout duration); Number of immobile phases/sleep period (Number of nocturnal sleep bouts);	Quetiapine significantly reduced the duration of night-time wake bouts. Both quetiapine and haloperidol had significant reductions in delusions and agitation. Quetiapine also significantly improved depression and anxiety, while haloperidol significantly worsened aberrant motor behaviour. Both quetiapine and haloperidol improved word recall, while quetiapine improved word-list memory and produced a non-significant overall increase in MMSE score.
Scherder [101]	1999	Netherlands	NH	16	81.7	Inclusion criterion: Diagnosis of probable Alzheimer's Dementia	RCT	Transcutaneous Electrical Nerve Stimulation	6 weeks	Sham TENS	Wrist actigraphy;	IS; IV; RA;	Significant improvement in inter-daily stability.
Scherder [106]	2003	Netherlands	NH	16	IG: 86.75, CG: 87.88;	IG: 17.88; CG: 20.38	RCT	Low frequency cranial electrostimulation;	6 weeks	Sham electrostimulatio n;	Wrist actigraphy;	IS; IV; RA; Salivary cortisol;	No significant effect on rest-activity rhythm.
Scherder [107]	2006	Netherlands	NH	20	IG: 83.7; CG: 84.5;	IG: 18.2; CG: 20;	RCT	High frequency cranial electrostimulation;	6 weeks	Sham electrostimulatio n;	Wrist actigraphy;	IS; IV; RA; Salivary cortisol;	No significant effect on rest-activity rhythm.
Schnelle [100]	1999	USA	NH	230	IG: 82.6)7.4); CG: 85.3(11.9);	IG: 11.1(9.4); CG: 10.7(9.1);	RCT Cluster	Noise, light and incontinence care intervention		Delayed intervention;	Wrist actigraphy; Nursing observation logs;	Wakes associated with noise; Wakes associated with light; Wakes associated with noise and light; Wakes (other); Night % sleep; Peak sleep duration; Average sleep bout duration;	No significant effect on sleep.
Scoralick [192]	2017	Brazil	Community	24	IG: 83.4(9.1); CG: 80.8(5.4);	IG: 10.6; CG: 11.9;	RCT	Mirtazapine 15mg	14 days	Placebo	Wrist actigraphy;	NTST; NNA; Night % sleep: WASO; DTST; Number of daytime naps;	No significant effect on night-time sleep. Significant increase in daytime sleep.
Scripnikov [244]	2007	Ukraine	Community	400		Inclusion criterion: Diagnosis of probable dementia	RCT	Ginkgo biloba Egb 761 240mg	22 weeks	Placebo	NPI	NPI-sleep;	Significant improvement in sleep (NPI-sleep domain) and behaviour (NPI).

Serfaty [161]	2002	UK	NH + community	44	84.2(7.6)	13.4(8.5)	RCT Crossover	Melatonin 6mg	2 weeks	Placebo	Wrist actigraphy; Sleep Evaluation Questionnaire (visual analogue); Caregiver log;	Sleep onset time; Wakening time; SE; TST; NNA; Sleep Evaluation Questionnaire score;	No significant effect on sleep.
Singer [159]	2003	USA	Community	157	77.4(8.9)	13.9(8.8)	RCT	Melatonin 2.5mg SR, 10mg IM;	8 weeks	Placebo	Wrist actigraphy; SDI;	NTST; DTST; DTST/NTST; WASO; SE; SDI; Sleep Quality Rating;	The Melatonin 2.5mg group saw significant improvement in caregiver rating of subjective sleep quality and in global NPI scores.
Sloane [85]	2015	USA	Community	17	65% were ≥80 years, while 35% were 65-79 years.	12.7(9.1)	RCT Crossover	Blue-white Bright Light	6 weeks	Yellow-red dim light	Wrist actigraphy; PSQI; Medical Outcomes Study Sleep Scale; ESS;	NTST; SoL; Night % sleep; Number of sleep bouts; IS; IV; PSQI; PSQI SE; ESS; Medical Outcomes Study Sleep Scale: total, sleep disturbance, sleep adequacy, daytime somnolence, sleep problems;	No significant change in sleep parameters. Significant improvement in caregiver burden (Zarit) and caregivers' subjective sleep quality (PSQI).
Song [171]	2014	S. Korea	Outpatients	92	IG: 68.5(11.3), CG: 70.2(7.4);	Donepezil morning: 19.0(4.8); Donepezil nocte: 18.8(5.5);	RCT	Donepezil 10mg (mane)	8 weeks	Donepezil nocte	Visual analogue scale - sleep quality;	Visual analogue scale - sleep quality	Switching donepezil to morning administration produced significant improvements in subjective sleep quality and daytime sleepiness.
Song [114]	2019	China	Community	120	75.78(6.28)	MoCA. IG: 22.03(1.81); CG: 22.1(1.92);	RCT	Aerobic exercise	16 weeks	Education programme	PSQI;	PSQI;	Significant improvements in cognition (MoCA), quality of life (QoL-AD), mood (GDS), and subjective sleep quality (PSQI)
Spagnolo [245]	1983	Italy	Hospital	30	78.93(1.16)	Inclusion criterion: Diagnosis of multi-infarct dementia or dementia with a predominantly vascular pathogenesis	RCT	Etoperidone 100mg	3 weeks	Thioridazine 75mg	Stuard Hospital Geriatric Rating Scale	Stuard Hospital Geriatric Rating Scale- sleep domain	Both groups showed significant within-group improvement in sleep.
Stefani [246]	2021	Austria & Spain	Outpatient clinic	36	71.3(6.36)	24.9(3.41)	RCT	Nelotanserin 80mg	4 weeks	Placebo	PSG	Number of clinically relevant RBD behaviours per 10 minutes of REM sleep;	No significant improvement in REM SBD.
Stotsky [247]	1984	USA	NH + hospital	610	76.67	All participants were stated to be senile	RCT	Thioridazine	4 weeks	Diazepam/Placebo	modified Hamilton Anxiety Scale	modified Hamilton Anxiety Scale - insomnia subdomain	Significant improvement in sleep and anxiety (modified Hamilton Anxiety Scale) compared to placebo.

Taheri [113]	2019	Iran	Community	68	IG: 67.3(2.1), CG: 68.1(2.4);	Inclusion criterion: cognitive impairment	RCT	Dynamic Sitting Exercise	2 weeks	Rest	Wrist actigraphy;	SE; WASO; Movement and fragmentation index;	Significant improvement in sleep efficiency, wakefulness after sleep onset, and movement and fragmentation index. Significantly improved performance on Stroop test.
Thodberg [134]	2016	Denmark	NH	124	Median: 85.5	Median: 14	RCT	Dog visit	6 weeks	Robotic seal; soft cat toy;	Wrist actigraphy;	NTST; SE; Sleep fragmentation index;	No significant improvement in sleep parameters.
Troussiere [129]	2014	France	Outpatient clinic	28	IG: Median 73.4; CG: Median 77.6;	IG: 23.5; CG: 20	Observational study	CPAP	4.1 years (mean)	Usual care	ESS	ESS	Significantly greater mean annual decline in cognition in the control group (MMSE).
Valtonen [248]	2005	Finland	NH	70	81(9)	8(8)	RCT Crossover	Night milk	8 weeks	Normal milk	Nursing log;	Sleep quality;	Significant within-group improvement in sleep quality in the group who consumed night milk during the Winter months. This effect was likely seasonal, however, as the control group had a similar improvement in sleep at the same time.
Van Someren [102]	1998	Netherlands	NH	19	84(1.5)	Inclusion criterion: Diagnosis of probable Alzheimer's Disease	RCT	Transcutaneous Electrical Nerve Stimulation	6 weeks	Sham TENS	Wrist actigraphy;	IS; IV; RA;	Significant improvement in inter-daily stability.
Wade [160]	2014	UK & USA	Outpatient Clinic	73	IG: 73.5(8.6); CG: 77.3(6.6);	IG: 22.1(3.5); CG: 21.4(4.7);	RCT	Melatonin 2mg	24 weeks	Placebo	PSQI	PSQI; PSQI domains	Significant improvements in subjective sleep efficiency (PSQI), cognition (MMSE) and function (IADL).
Wang [183]	2020	China	Outpatient Clinic	40	IG: 62.65(12.57), CG: 63.14(11.65);	MoCA: IG: 17.03(4.53); CG: 17.7(4.29);	RCT	Trazadone 50mg	4 weeks	Placebo	PSG; PSQI; ESS; Fatigue Scale (FS-14);	SoL; NTST; SE; WASO; %N1; %N2; %N3; %REM; Arousal Index; AHI; Apnoea Index; PSQI; ESS;	Significant increase in sleep efficiency and NREM stage 3 percentage, with significant decrease in wakefulness after sleep onset, NREM stage 1 percentage and arousal index. Significant improvement in subjective sleep quality (PSQI) and daytime sleepiness (ESS). Significant improvement in anxiety (HAMA) and in concentration and recall (MoCA).

Wang [249]	2022	USA & Canada	Community	344	72.6	Inclusion criteria: Diagnosis of dementia with MoCA 10-23	RCT	Mevidalen 10/30/75mg	12 weeks	Placebo	Wrist actigraphy; ESS;	DTST; NTST; ESS;	Significant decrease in daytime total sleep time for all doses of mevidalen during treatment, with significant increase in night-time total sleep time post-treatment in the 75mg group.
Wang, Lei [103]	2022	China	Community	60	IG: 66.9(3.36); CG: 67.0(4.36);	Inclusion criterion: Diagnosis of MCI	RCT	Transcutaneous auricular vagus nerve stimulation	24 weeks	Sham TaVNS	PSQI; ESS; Rapid Eye Movement Sleep Behaviour Disorder Screening Questionnaire ;	PSQI; ESS; Rapid Eye Movement Sleep Behaviour Disorder Screening Questionnaire;	Significant improvement in cognition (MoCA), with significant within-group improvements in subjective sleep quality, daytime sleepiness (ESS), and functional ability (FAQ).
Wang, Lina [115]	2020	China	Community	116	IG: 68.37(5.27), CG: 68.24(5.15);	MoCA. IG: 21.65(2.22); CG: 21.41(2.41);	RCT	Structured Limbs Exercise Program	24 weeks	Wait list	PSQI;	PSQI	Significant improvement in subjective sleep quality (PSQI), mood (GDS), and cognition (MoCA).
Weise [144]	2019	Germany	NH	20	85.05(5.93)	Inclusion criterion: Diagnosis of dementia	RCT	Individualised music intervention	4 weeks	Wait list	Visual analogue scale - sleep quality;	Visual analogue scale - sleep quality	Significant improvement in sleep quality.
Yang [139]	2017	China	Community	39	IG: 62.94(5.45); CG: 64.23(5.72);	IG: 21.26(3.05); CG: 22.0(2.78)	RCT	Tai Chi - Group session	13 weeks	Tai Chi - Individual session	Parkinson's Disease Sleep Scale;	Parkinson's Disease Sleep Scale Total;	Both groups showed significant within-group improvement in sleep and non-motor symptoms of Parkinson's Disease, whilst the intervention group also showed significant improvement in cognition.
Yang [250]	2022	China	Outpatient Clinic	48	73(6.9)	23 participants were stated to have MCI and 25 participants were stated to have mild Alzheimer's Dementia.	RCT	Sports Stacking	12 weeks	Usual care	PSQI;	PSQI;	No significant effect on sleep. Significant improvement in Auditory Verbal Learning Test.
Yehuda [251]	1996	Israel	Community	100	Range: 50-73 years	7.8(3.8)	RCT	SR-3(fatty acids)	4 weeks	Placebo	Bespoke question- sleep problems?	Bespoke question - sleep problems?	Some improvement in sleep.

Yin [162]	2015	China	Community	156	77.05	IG: 17.31(3.06); CG: 18.13(2.99);	Controlled trial	4 Groups: Donepezil plus: Risperidone 0.5-1mg/Zolpidem 5-10mg/Melatonin 2.55mg/No drug.	5 years	Donepezil	ESS; PSQI;	ESS; PSQI;	Risperidone had significantly improved subjective sleep quality (PSQI) and significantly lower daytime sleepiness (ESS) compared to all three other groups, and significantly reduced neuropsychiatric disturbances (NPI) and institutionalisation compared to no drug. Zolpidem had significantly improved subjective sleep quality (PSQI) and significantly lower daytime sleepiness (ESS) compared to the no drug group.
Yu [116]	2022	Hong Kong	Community	50	CG: 63.7(4.7); M1: 63.5(7.0); M3: 63.5(5.7); V1: 63.4(5.2); V3: 63.3(5.1);	CG: 19.9(3.5); M1: 19.7(1.9); M3: 18.9(1.9); V1: 20.1(1.7); V3: 19.6(2.5);	RCT	Moderate intensity walking once per week (M1)/Moderate intensity walking thrice per week (M3)/Vigorous intensity walking once per week (V1)/Vigorous intensity walking thrice per week (V3);	12 weeks	Stretching	PSQI;	PSQI	Significant improvements in cognition (MoCA) and anxiety (HADS) but no significant change in sleep quality.
Zhou [108]	2022	China	Community	70	IG: 70; CG: 74;	Inclusion criterion: Diagnosis of Alzheimer's Dementia.	RCT	Transcranial magnetic stimulation	4 weeks	Sham TMS	PSQI;	PSQI	Significant improvement in subjective sleep quality (PSQI) and cognition (ADAS-Cog).
Zhu [140]	2020	China	Hospital and outpatient clinic	41	IG: 68.53(1.90), CG: 67.77(1.72);	IG: 21.37(2.52); 22.05(2.78);	RCT	Tai Chi	12 weeks	Routine exercise	Parkinson's Disease Sleep Scale;	Parkinson's Disease Sleep Scale Total;	Significant improvement in sleep and cognition (MoCA).

Table S2. Characteristics of included trials. *NH=nursing home; IG=intervention group; CG=control group; RCT=Randomised controlled trial; TIB=time in bed; DTIB=day time in bed; NTIB=Night time in bed; TST=total sleep time; NTST=night-time total sleep time; DTST=daytime total sleep time; SE=sleep efficiency; WASO=wakefulness after sleep onset; NNA=number of night-time awakenings; SoL=sleep onset latency; TWT=total wake time; NTWT=night-time total wake time; DTWT=day total wake time; NREM=non-rapid eye movement; REM=rapid eye movement; DLMO=dim light melatonin onset; IS=inter-daily stability; IV=intra-daily variability; RA=relative amplitude; MESOR=midline estimated statistic of rhythm; AHI=apnoea-hypopnoea index; RBD=REM sleep behaviour disorder; TMS=Transcranial Magnetic Stimulation; DCS=Direct Current Stimulation; PSQI=Pittsburgh Sleep Quality Index; SDI=Sleep Disorders Inventory; ISI=Insomnia Severity Index; AIS=Athens Insomnia Scale; ESS=Epworth Sleepiness Scale; NPI=Neuropsychiatric Inventory; GDS=Geriatric Depression Scale; SAS=Self-rating Anxiety Scale; PSS=Perceived Stress Scale; HAMA=Hamilton Anxiety Rating Scale; HAMD=Hamilton Depression Rating Scale; HADS=Hospital Anxiety & Depression Scale; CSDD=Cornell Scale for Depression in Dementia; CMAI=Cohen-Mansfield Agitation Inventory; FAQ=Functional Activities Questionnaire; BEHAVE-AD=Behavioural Pathology in Alzheimer's Disease Rating Scale; QoL-AD=Quality of Life in Alzheimer's Disease; QUALID=Quality of Life in Late-Stage Dementia; MMSE=Mini-Mental State Examination; MoCA=Montreal Cognitive Assessment; WAIS=Weschler Adult Intelligence Scale; CDR=Clinical Dementia Rating Scale; PSG=Polysomnography; CPAP=Continuous Positive Airway Pressure.*
