# Supplementary material for: Pharmacological and Non-Pharmacological Interventions to Improve Sleep in People with Cognitive Impairment: A Systematic Review and Meta-Analysis
Source: Int J Environ Res Public Health. 2025 Jun 18;22(6):956. doi: 10.3390/ijerph22060956 (PMC12192850; doi:10.3390/ijerph22060956)
Supplement: Supplementary file 1 [file Figure S2.pdf › Figure S2.pdf]

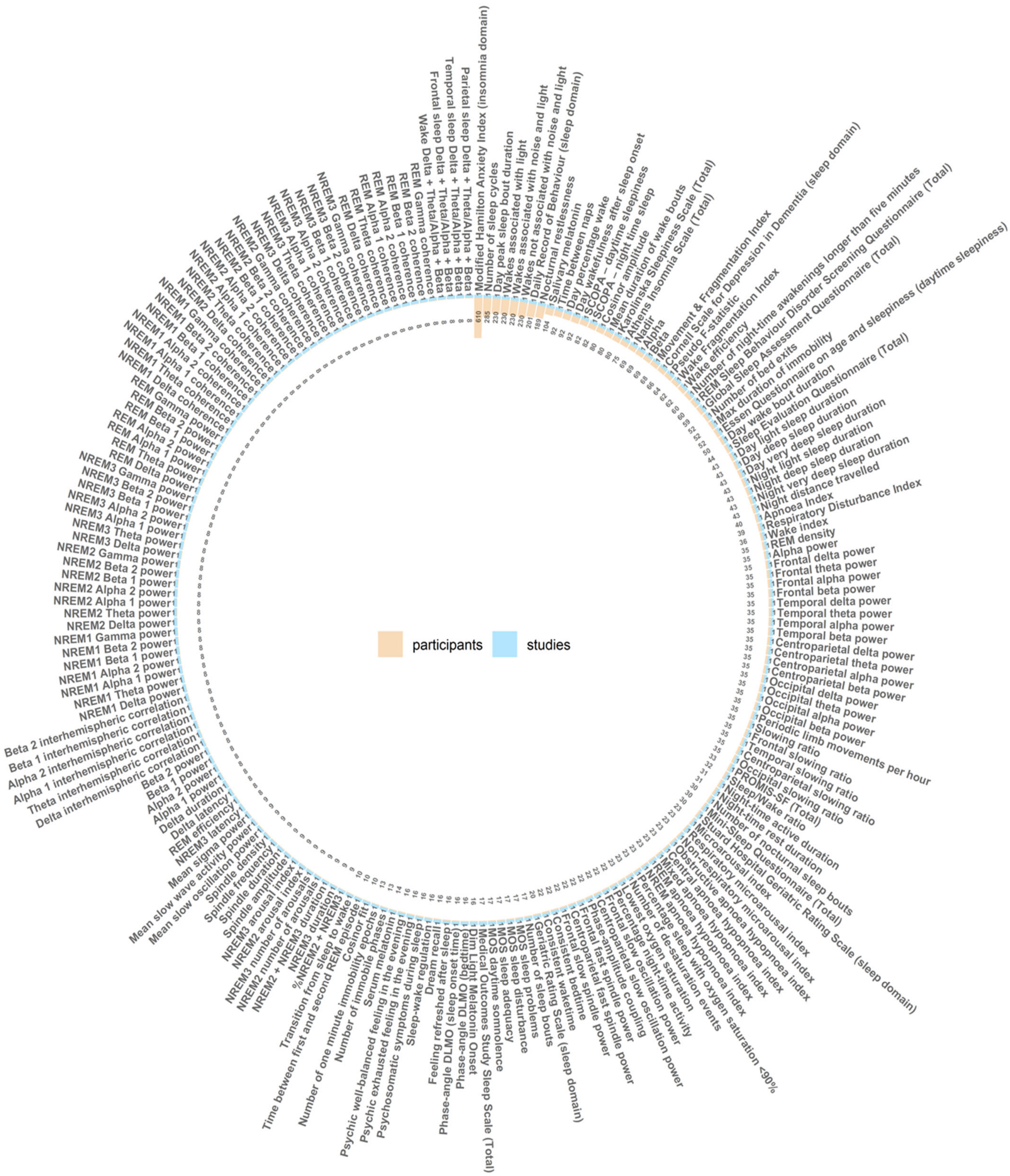

Figure S2. The sleep outcome measures reported in only one trial (n=205), including the number of trials each sleep outcome measure was reported in and the total number of participants involved in those trials.
